# Controls on the isotopic composition of microbial methane

**DOI:** 10.1126/sciadv.abm5713

**Published:** 2022-04-06

**Authors:** Jonathan Gropp, Qusheng Jin, Itay Halevy

**Affiliations:** 1 Department of Earth and Planetary Sciences, Weizmann Institute of Science, Rehovot, Israel.; 2 Department of Earth Sciences, University of Oregon, Eugene, OR, USA.

## Abstract

Microbial methane production (methanogenesis) is responsible for more than half of the annual emissions of this major greenhouse gas to the atmosphere. Although the stable isotopic composition of methane is often used to characterize its sources and sinks, strictly empirical descriptions of the isotopic signature of methanogenesis currently limit these attempts. We developed a metabolic-isotopic model of methanogenesis by carbon dioxide reduction, which predicts carbon and hydrogen isotopic fractionations, and clumped isotopologue distributions, as functions of the cell’s environment. We mechanistically explain multiple isotopic patterns in laboratory and natural settings and show that these patterns constrain the in situ energetics of methanogenesis. Combining our model with data from environments in which methanogenic activity is energy-limited, we provide predictions for the biomass-specific methanogenesis rates and the associated isotopic effects.

## Teaser

Model of hydrogenotrophic methanogenesis explains how enzyme level activity determines methane isotopic composition as function.

## INTRODUCTION

Methane (CH_4_) is a major greenhouse gas, with both natural and anthropogenic sources (*1*). The primary natural source of biogenic methane emissions is archaeal methanogenesis in anoxic environments (*2*), about a third of which is hydrogenotrophic (*3*), with the net reaction

$$4H_{2}+CO_{2}⇄CH_{4}+2H_{2}O$$ (1)

Strong isotopic discrimination during biological and abiotic methane formation has motivated the use of methane hydrogen and carbon isotopes to trace its production and consumption processes, construct global methane budgets, and evaluate its climatic impacts (*1*, *4*, *5*). In early studies of the isotopic composition of methane in the environment, temperature-dependent thermodynamic isotopic equilibrium between methane and carbon dioxide was considered to be an important control on methane carbon isotopes (*6*–*9*). Contemporaneous laboratory culture experiments, in contrast, displayed methane carbon and hydrogen isotope compositions that departed from thermodynamic isotopic equilibrium (*10*–*14*). Subsequent experimental studies that empirically related the thermodynamics of microbial methanogenesis (the Gibbs free energy of the net reaction) to the associated isotopic discrimination offered a means to reconcile the early laboratory and environmental results (*15*–*21*). Organism-level models that use isotopic mass balance to explain the observed range of microbial isotopic discrimination in hydrogenotrophic methanogenesis have been developed (*22*–*24*), but to date, these models have prescribed rather than resolved the microbial biochemistry. It has been difficult, therefore, to distinguish between different methane sources, different modes and extents of environmental methane cycling, and different environmental controls on the microbial isotopic discrimination as drivers of observed variations in the isotopic composition of methane.

Metabolic-isotopic models link the thermodynamics and kinetics of biochemical reactions to the isotopic discrimination imparted by metabolic activity (*25*). To constrain the microbial component of observed variations in the carbon and hydrogen isotope composition of methane, we developed and analyzed a full metabolic-isotopic model of hydrogenotrophic methanogenesis. The model predicts the isotopic discrimination and its relation to the energy available to drive the methanogenesis reaction and to cell-specific methanogenesis rates (csMRs) in laboratory cultures, providing a quantitative tool to explore the dependence of methane isotopic composition on the metabolic state of the methanogenic cells. Extending our analysis to energy-limited conditions, which are prevalent in natural environments, our model predicts the environmental and metabolic controls on the isotopic composition of methane.

## RESULTS AND DISCUSSION

### Calibration and validation of a metabolic model of hydrogenotrophic methanogenesis

**Fig. 1.**
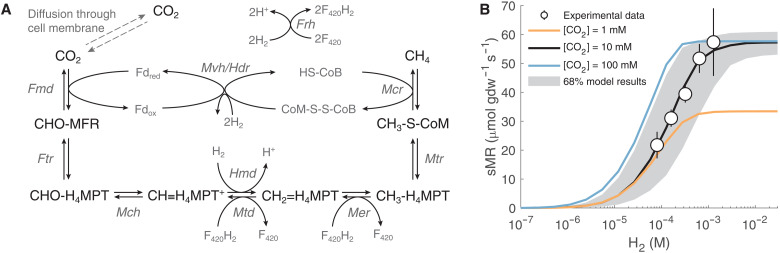
A metabolic model of hydrogenotrophic methanogenesis. (**A**) Schematic illustration of the metabolic pathway including all reactions that are included in the metabolic model. Abbreviated enzyme names are in italics (table S1). (**B**) Metabolic model calibration to measured biomass-specific methanogenesis rate (sMR) as a function of the H_2_ concentration. Measured biomass-specific methanogenesis rate (circles) during the linear and stationary phase in laboratory cultures of *M. thermoautotrophicus* grown at 60°C (error bars are SDs for four experiments) (*66*). The median (solid lines) of 10^3^ model simulations with 1, 10, and 100 mM [CO_2_] and 68% (gray envelope) of simulations with 10 mM [CO_2_] are shown. The envelope represents *K*_M_ values that were drawn from posterior distributions resulting from the model calibration (see Methods, fig. S2, and table S5).

We constructed mass balance equations for the concentrations of the intracellular metabolites in the hydrogenotrophic methanogenesis pathway (Fig. 1A). In such a mass balance, enzymatically catalyzed reactions in the pathway produce and consume the various metabolites at rates that are determined by reversible Michaelis-Menten–type rate laws, which account for the reaction kinetics and thermodynamics (details in Methods and tables S1 and S2) (*26*). We regard the “transformed” standard-state and actual Gibbs free energies ($\Delta {G^{\prime }}_{r}^{0}$ and ΔG_r_, respectively), which are calculated at the constant pH and ionic strength that are maintained by intracellular homeostasis (*27*). Given fixed extracellular aqueous concentrations of CO_2_, H_2_, and CH_4_ (henceforth denoted [CO_2_], [H_2_], and [CH_4_]) and pH, which define the Gibbs free energy of net methanogenesis (ΔG_net_), the mass balance equations are solved for the steady-state concentrations of the intracellular metabolites (fig. S1). At this steady state, the model links metabolite concentrations to the net rate of methanogenesis and to the gross forward and reverse rates of the individual reactions.

Our model specifically represents methanogens that do not have membrane-associated methanophenazines. These methanogens characteristically couple ferredoxin (Fd) reduction by H_2_ to the reduction of the heterodisulfide CoM-S-S-CoB by the [NiFe]-hydrogenase and the heterodisulfide reductase enzyme complex (Mvh/Hdr, table S1) (*28*). Recent observations suggest that Mvh/Hdr may form a larger complex with formylmethanofuran dehydrogenase (Fmd) and that formate or coenzyme F_420_ may serve as the electron donor for this coupled reaction instead of soluble Fd (*29*). We did not represent this complex in the model, as the physiological conditions under which it emerges are not yet clear. Should this complex prove to be of ecological significance, it may readily be included in future versions of our model.

We calibrated our metabolic model to available measurements of biomass-specific methanogenesis rates and their relation to [H_2_] in the micromolar to millimolar range (Fig. 1B). The procedure for calibrating the metabolic model parameters to obtain optimal model-measurement agreement is described in the Methods. The enzyme maximal rate capacities (*V*^+^) required for the model are taken from crude cell extract measurements (i.e., in vitro; table S2). We found that over the range of [H_2_] measured in culture experiments, optimal model agreement with experimental in vivo results requires scaling in vitro rate capacities up by a factor of ≈11 (fig. S2J). The necessity for this scaling may reflect different enzyme concentrations in the cell extracts compared to living cells, product buildup, and substrate depletion, among other factors (*25*). After model calibration to reproduce [H_2_]-dependent biomass-specific methanogenesis rates, we next validated the model (with no further parameter tuning) against available measurements of metabolite concentrations and oxidation states.

The metabolic model accounts for electron transfer from H_2_ to the intermediate metabolites through three electron carriers, F_420_, coenzyme B (HS-CoB), and Fd, thereby providing insight into the oxidation state of the cell. We validated the model’s predictions for the oxidation state of these electron carriers against available measurements (table S3). The reduction of F_420_ depends directly on [H_2_], and we find that F_420_ in the model is 50% reduced at [H_2_] of ≈50 μM (fig. S1K). This [H_2_] is higher than observed in cell suspensions (50% reduction at [H_2_] of ≈5 μM; table S3) but in line with observations in fed-batch cultures (F_420_ at 65 to 90% reduced at a H_2_ concentration of 10 to 40 μM) (*30*). We note that in the model, the degree of F_420_ reduction is insensitive to variations in the kinetic parameters (*V*^+^; and the Michaelis constants, *K*_M_) and is only sensitive to the $\Delta {G^{\prime }}_{r}^{0}$ of the F_420_-reducing [NiFe]-hydrogenase (Frh)-catalyzed reaction, which was calculated using the standard reduction-oxidation potential (E′^0^) of the reaction F_420_ + 2*e*^−^ → F_420_H_2_ (table S4). We found that setting a $\Delta {G^{\prime }}_{r}^{0}$ value that is 7 kJ mol^−1^ more negative than the default model value results in a better match with the cell suspensions. However, this implies an E′^0^ value for F_420_ of −320 mV, which is substantially less negative than available measurements (−373 to −353 mV) (*31*–*33*), and we adopt the $\Delta {G^{\prime }}_{r}^{0}$ calculated with the more reasonable F_420_ E′^0^ value. The model HS-CoB/CoM-S-S-CoB couple is 2 to 20% reduced at [H_2_] of 10 μM, depending on CO_2_ concentrations (fig. S1L), within the observed range of this couple’s oxidation state at similar [H_2_] (0.5 to 55% reduced; table S3) (*34*). We note that over the range of [H_2_] at which the electron carrier oxidation state measurements were made, ΔG_net_ is negative enough that some of the reactions in the pathway operate in disequilibrium. Model-measurement agreement, therefore, suggests that both the thermodynamic and kinetic model parameters have approximately correct values.

Our observation-validated model predictions suggest that the intracellular oxidation state of electron carriers generally does not reflect electrochemical equilibrium and is instead maintained dynamically by oxidation and reduction fluxes that depend on the cell’s metabolic activity. This dynamic control means that measurements of the oxidation state of specific reduction-oxidation couples do not necessarily bear on the oxidation state of other reduction-oxidation couples (both inorganic and organic) and on the overall oxidation state of the cell.

We also validated the model predictions against the few available measurements of metabolite concentrations. Concentrations of HS-CoM in the model vary as a function of both [H_2_] and [CO_2_] (fig. S1I), spanning a wide range between 3 and 200 μM at [H_2_] of 10 μM and [CO_2_] between 0.1 and 100 mM. Measured HS-CoM concentrations show a very similar range, 4 to 200 μM, at similar [H_2_] (table S3) (*35*). Measured methenyl tetrahydromethanopterin (CH-H_4_MPT) concentrations were 13 to 35 μM at [H_2_] of ≈1.5 mM under different growth regimes (*35*), in agreement with model CH-H_4_MPT concentrations of ≈10 to 100 μM at [H_2_] of 1.5 mM. Last, the ratio of formyl methanofuran (CHO-MFR) to methanofuran (MFR) in the model ranges between 1 and 2.6 at [H_2_] of 10 μM, within the lower range of this ratio measured under similar conditions (1.15 to 10; table S3) (*34*). As in the case of the oxidation state of electron carriers, model-measurement agreement in the case of metabolite concentrations suggests that both the thermodynamic and kinetic model parameters have broadly correct values.

**Fig. 2.**
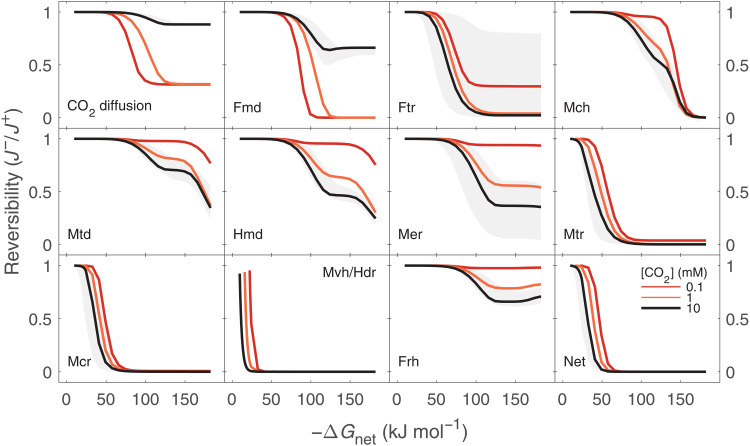
Reversibility of reactions in hydrogenotrophic methanogenesis. Reaction reversibility ($J_{i}^{-}/J_{i}^{+}$) against ΔG_net_. The short names of the enzymes are in accordance with Fig. 1A and table S1. The “Net” reversibility shown in the bottom right is the CO_2_-CH_4_ reversibility, which is determined as the product of the individual reversibilities of the reactions in the carbon pathway (Fmd to Mcr). The median (black line) and 68% (gray envelope) of 10^3^ model simulations with 10 mM [CO_2_] are shown. The envelope represents *K*_M_ values that were drawn from posterior distributions resulting from the model calibration (see Methods, fig. S2, and table S4). The medians of simulations with [CO_2_] of 0.1 and 1 mM are shown in red and orange lines, respectively. Simulations were carried out at [H_2_] of 1 nM to 10 mM and [CH_4_] of 10 μM.

The calibrated and validated biochemical model forms the basis for the metabolic-isotopic coupling. As ΔG_net_ becomes increasingly negative (e.g., with increasing [H_2_]), the individual reactions in the pathway depart from equilibrium to various extents (Fig. 2). We quantify the departure from equilibrium of individual reaction *i* by the reaction reversibility, defined as the ratio of the reverse to forward gross rates of that reaction and related to its actual Gibbs free energy (ΔG_r,*i*_) (*26*, *36*, *37*)

$$J_{i}^{-}/J_{i}^{+}=\text{exp}\;(\Delta G_{r,i}/\mathrm{RT})$$ (2)

where R is the gas constant (in kilo joules per mole per kelvin), T is the temperature (in kelvin), and $\Delta G_{r,i}=\Delta G_{r,i}^{\prime 0}+\mathrm{RT}\;\text{ln}\;Q$ [in kilo joules per mole (kJ mol^−1^); where *Q* is the reaction quotient]. We henceforth omit the subscripted *i* from the individual reaction $\Delta {G^{\prime }}_{r}^{0}$ and ΔG_r_.

For net forward methanogenesis rates, the reversibility ($J_{i}^{-}/J_{i}^{+}$) of individual reactions varies between zero (a near-unidirectional, kinetically controlled reaction) and unity (a near-equilibrium reaction). The uncertainty in reaction reversibility (gray envelopes in Fig. 2) is due to the unknown *K*_M_ values, which are drawn from distributions produced in the metabolic model calibration process (see Methods and fig. S2). Some reactions do not become completely irreversible ($J_{i}^{-}/J_{i}^{+}\to 0$) over the explored ΔG_net_ range (e.g., the Frh-catalyzed reduction of F_420_), whereas others depart from a reversibility of unity at ΔG_net_ as modestly negative as −15 kJ mol^−1^ (Mvh/Hdr-catalyzed reaction) and −50 kJ mol^−1^ [methyl-coenzyme M reductase (Mcr) and H_4_MPT S-methyltransferase (Mtr)-catalyzed reactions] and become near irreversible. We note that net methane production in our model is only possible at ΔG_net_ values more negative than ≈−10 kJ mol^−1^, a threshold below which adenosine triphosphate (ATP) production is not possible (see Methods). The differential response of the individual reaction reversibilities to changes in ΔG_net_ is a combined function of the reaction thermodynamics ($\Delta {G^{\prime }}_{r}^{0}$) and the enzyme kinetics, specifically the *V*^+^ and *K*_M_ values. Neither $\Delta {G^{\prime }}_{r}^{0}$ nor *V*^+^ and *K*_M_ in isolation predict the pattern of differential departure from equilibrium; coupled thermodynamics and kinetics must be considered.

### An isotopic model calibrated to laboratory cultures

The isotopic discrimination between substrate (*s*) and product (*p*) is described by the isotopic fractionation factor *^r^*α_*s*-*p*_ = *^r^*R*_s_*/*^r^*R*_p_*, where ^13^R = ^13^C/^12^C, ^2^R = D/H (with D ≡ ^2^H and H ≡ ^1^H), and “*r*” denotes the rare isotope. The net isotopic fractionation expressed in an individual (bio)chemical reaction may vary between thermodynamic equilibrium and kinetic end-members, associated respectively with a reversible reaction and unidirectional forward reaction to form the reaction product. The equilibrium isotopic fractionation factor (EFF) between *s* and *p* is denoted by $\alpha_{s-p}^{\text{eq}}$, and the kinetic fractionation factors (KFFs) are denoted by α^+^ or α^−^ for the forward and reverse direction of the reaction, respectively. The EFFs are related to the KFFs of the respective reaction by $\alpha_{s-p}^{\text{eq}}=\alpha^{-}/\alpha^{+}$. Normal KFFs, where the isotopically heavy substrate reacts more slowly than the isotopically light substrate, are, by definition, smaller than unity. As most α values are close to unity, we use the ε notation for convenient comparison of fractionations [ε = 1 − α, in per mil (‰)].

**Fig. 3.**
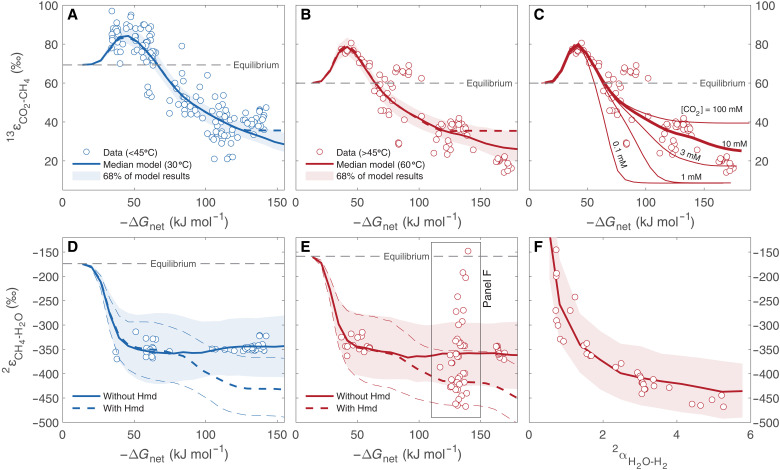
Model-laboratory culture comparison of bulk carbon and hydrogen isotope fractionation. Mesophilic (30° to 40°C; blue circles) and thermophilic (≥55°C; red circles) experimental data and model results (lines and envelopes) at 30°C (blue) and 60°C (red) and the equilibrium isotopic fractionation (dashed lines). (**A**, **B**, **D**, and **E**) ^13^ε_CO_2_-CH_4__ and ^2^ε_CH_4_-H_2_O_ against ΔG_net_. The envelopes represent 68% of 10^3^ model simulations, with KFFs drawn from calibrated distributions (see Methods and fig. S3). (**C**) ^13^ε_CO_2_-CH_4__ against ΔG_net_ for different extracellular CO_2_ concentrations ([CO_2_]; shown next to the corresponding model curve). (**F**) Mixing effects on ^2^ε_CH_4_-H_2_O_ as a function of the H_2_O-H_2_ isotopic fractionation (^2^α_H_2_O-H_2__), compared to laboratory culture data at 60°C (*20*). The envelope represents 68% of 10^3^ model simulations, with KFFs drawn from calibrated distributions.

With the forward and reverse gross fluxes calculated in the metabolic model and with values assigned to the EFFs and KFFs of the individual reactions, an isotopic mass balance may be solved for the net isotopic fractionation between the pathway substrates and products (*25*, *38*). The uncertainty on calculated EFFs is relatively small (*39*), while most KFFs in hydrogenotrophic methanogenesis are unknown, and we drew their values from uniform distributions (see Methods). Model ΔG_net_–fractionation relations calculated with the randomly drawn KFF values were compared to experimental data and were used to generate distributions of the KFF values that minimize model-measurement mismatch (see Methods and fig. S3). The ranges of KFF values calibrated in this way are the main source of uncertainty in the isotopic model (i.e., main reason for the uncertainty envelopes in Fig. 3). We note that existing CH_4_-H_2_O hydrogen isotope fractionations from culture experiments conducted under similar conditions differ by as much as ≈100‰ at a given ΔG_net_ value [e.g., (*20*, *40*); fig. S4]. We calibrated the hydrogen isotope KFFs against a curated dataset, which includes only a subset of the experimental data (see the Supplementary Materials), and tested the effects of this choice on the posterior KFF distributions. We found relative insensitivity to the choice of calibration dataset, other than for the KFF of Mvh/Hdr, which has smaller (i.e., less negative) best-fit values when the calibration includes all of the experimental data (fig. S5) instead of the curated dataset. All other inferences made on the basis of the calibrated model are robust to the choice of calibration dataset.

After calibration of the carbon and hydrogen isotope KFFs to experimental data, we used the model (with no further tuning of the isotopic parameters) to determine the relations between ΔG_net_ and the abundances of doubly substituted (“clumped”) methane isotopologues, which have yet to be systematically explored in experiments.

### Bulk carbon isotope fractionation between CO_2_ and CH_4_

In laboratory cultures, the CO_2_-CH_4_ carbon isotope fractionation (^13^ε_CO_2_-CH_4__) is inversely related to ΔG_net_ (fig. S4A) (*16*–*21*). At near-zero ΔG_net_, the individual reactions in the pathway operate close to equilibrium ($J_{i}^{-}/J_{i}^{+}\to 1$), and our model predicts ^13^ε_CO_2_-CH_4__ close to the temperature-dependent isotopic equilibrium fractionation (ε13CO2‐CH4eq). When ΔG_net_ becomes slightly negative, ^13^ε_CO_2_-CH_4__ peaks to larger-than-equilibrium values of 80 to 100‰ at ΔG_net_ of ≈−45 kJ mol^−1^, followed by a gradual decline to ≈30‰ reached at ΔG_net_ of ≈−120 kJ mol^−1^ (Fig. 3, A and B). These larger-than-equilibrium ^13^ε_CO_2_-CH_4__ values have been observed in several experimental and environmental datasets and have been suggested to reflect a switch from an equilibrium to a kinetic fractionation in one of the network steps, with a KFF that is larger than the EFF (*41*). Our model reveals that this ΔG_net_–^13^ε_CO_2_-CH_4__ relation is indeed controlled by the landscape of combined departure from equilibrium of several reactions in the pathway. The carbon reaction network in methanogenesis is linear. In these networks, near-unidirectionality of an individual reaction ($J_{i}^{-}/J_{i}^{+}\to 0$) leads to expression of that reaction’s KFF and suppresses isotopic fractionation associated with downstream reactions (*25*). The Mcr- and Mtr-catalyzed reactions depart from equilibrium simultaneously at ΔG_net_ of ≈−25 kJ mol^−1^, and our model identifies the peak in ^13^ε_CO_2_-CH_4__ to reflect a weighted sum of these specific enzymes’ KFFs and the sum of EFFs of the upstream reactions (Fig. 2). The experimentally measured KFF of Mcr (−38‰) (*42*) is larger than its EFF (1‰) (*43*) and so is the model-predicted KFF of Mtr (≈−28‰) relative to its EFF (15‰) (*43*). Hence, partial expression of these KFFs results in the larger-than-equilibrium ^13^ε_CO_2_-CH_4__ at modestly negative ΔG_net_ values. The ^13^ε_CO_2_-CH_4__ floor at ΔG_net_ more negative than ≈−120 kJ mol^−1^ (Fig. 3, A and B) is defined by partial expression of the KFFs of formyltransferase (Ftr) and Fmd and suppression of the isotopic fractionations associated with downstream reactions.

At large negative ΔG_net_ values, ^13^ε_CO_2_-CH_4__ is also sensitive to the extracellular partial pressure of CO_2_ (*P*co_2_). At a steady state, intracellular CO_2_ utilization is exactly matched by net CO_2_ diffusion across the membrane. This net diffusive flux is the difference between large gross fluxes (into and out of the cell) when *P*co_2_ is high and smaller gross fluxes at lower *P*co_2_. Thus, the reversibility of net diffusion is low at low *P*co_2_ ($J_{i}^{-}/J_{i}^{+}\to 0$), and suppression of downstream net carbon isotopic fractionation results in small ^13^ε_CO_2_-CH_4__ (also referred to as a “reservoir effect”). The dependence of ^13^ε_CO_2_-CH_4__ on *P*co_2_ explains the smallest net fractionations observed in laboratory cultures (Fig. 3C) (*18*), as well as the dependence of ^13^ε_CO_2_-CH_4__ on pH in hyperalkaline settings (*44*, *45*).

### Carbon isotope fractionation between CO_2_ and a biomass precursor

We compared the results of the calibrated metabolic-isotopic model to measurements of the CO_2_-biomass carbon isotope fractionation. About a third of the carbon in the biomass of hydrogenotrophic methanogens derives from CH_3_-H_4_MPT produced in the methanogenesis pathway, ∼5% of which is directed to biomass production in laboratory cultures of methanogens that have no membrane-associated methanophenazines (*46*). In natural environments, and especially under energy limitation, the fraction of CH_3_-H_4_MPT directed to form biomass may be substantially smaller. Hence, we assumed that this biomass shunt has only a minor effect on the net CO_2_-CH_4_ carbon isotope fractionation and did not explicitly include it in our model. Another third (approximately) of the biomass carbon derives from reduction of CO_2_ to CO and the subsequent condensation of a carbonyl moiety with the CH_3_-H_4_MPT–derived methyl moiety to form acetyl-CoA (*47*). The last third (approximately) of the carbon in hydrogenotrophic methanogen biomass comes from various carboxylation reactions (*48*).

Within the above framework, the carbon isotope composition of biomass can be expressed as a function of the isotopic compositions of CO_2_ and CH_3_-H_4_MPT and a composite isotopic fractionation, which represents an appropriately weighted combination of the fractionations associated with the various biosynthesis reactions (eq. S3). The model carbon isotope fractionation between CO_2_ and CH_3_-H_4_MPT (^13^ε_CO_2_-CH_3__) covaries with ^13^ε_CO_2_-CH_4__ with a slope of ≈0.95 (fig. S6). With a few exceptions, observed carbon isotope fractionations between CO_2_ and biomass (^13^ε_CO_2_-B_) (*49*–*51*) covary with ^13^ε_CO_2_-CH_4__ along a scattered array with a slightly shallower slope (≈0.73; *R*^2^ = 0.72), which is offset from the model ^13^ε_CO_2_-CH_3__-^13^ε_CO_2_-CH_4__ curve by ≈10 to 30‰ (fig. S6). Isotopic mass balance requires that to maintain this approximately constant offset (≈20± 10‰) between the ^13^ε_CO_2_-CH_3__-^13^ε_CO_2_-CH_4__ curve and the observed ^13^ε_CO_2_-B_-^13^ε_CO_2_-CH_4__ array, the composite carbon isotope fractionation associated with biosynthesis reactions (e.g., CO_2_ reduction, acetyl-CoA condensation, and carboxylation) must covary with ^13^ε_CO_2_-CH_4__ (eq. S3). That is, with increasingly negative ΔG_net_, as ^13^ε_CO_2_-CH_4__ departs from CO_2_-CH_4_ carbon isotope equilibrium and gradually decreases toward the KFFs of Ftr and Fmd, so does the composite biosynthesis fractionation depart from isotopic equilibrium toward a kinetically controlled fractionation. This implies back reaction in at least some of the biosynthesis reactions, although we cannot confidently identify which reactions.

### Bulk hydrogen isotope fractionation between CH_4_ and H_2_O

In the case of hydrogen isotopes, we express the CH_4_-H_2_O fractionation (^2^ε_CH_4_-H_2_O_) rather than the H_2_O-CH_4_ fractionation, which is opposite in sign. This choice maintains a common sense of departure from equilibrium, in which both ^13^ε_CO_2_-CH_4__ and ^2^ε_CH_4_-H_2_O_ values decrease with increasingly negative ΔG_net_. In laboratory cultures, ^2^ε_CH_4_-H_2_O_ is ≈200‰ more negative than the temperature-dependent isotopic equilibrium fractionation (ε2CH4‐H2Oeq), and existing observations suggest that it does not display a clear dependence on ΔG_net_ (fig. S4B) (*16*, *19*, *20*, *40*, *52*). Unlike the linear carbon reaction network, the hydrogen reaction network has four branches, each of which has the potential for hydrogen atom exchange between pathway intermediates and H_2_O. Therefore, departure from equilibrium of one of the hydrogen atom exchange reactions does not preclude CH_4_-H_2_O hydrogen isotopic equilibrium. Specifically, the Mvh/Hdr-catalyzed reaction is near irreversible at ΔG_net_ values as high as −25 kJ mol^−1^ (Fig. 2), yet at this ΔG_net_ value, ^2^ε_CH_4_-H_2_O_ is approximately equal to ε2CH4‐H2Oeq (Fig. 3, D and E), and this arises from the high reversibility of the other hydrogen atom exchange reactions in the pathway. Only when the Mcr- and Mtr-catalyzed reactions sufficiently depart from reversibility (at ΔG_net_ ≤ −30 kJ mol^−1^), cutting off methane and HS-CoB from exchange with upstream intermediates that are close to hydrogen isotope equilibrium with H_2_O, does ^2^ε_CH_4_-H_2_O_ depart from ε2CH4‐H2Oeq (Fig. 3, D and E). At ΔG_net_ more negative than −40 kJ mol^−1^, the KFFs of Mcr, Mtr, and Mvh/Hdr control ^2^ε_CH_4_-H_2_O_ (fig. S3). In addition to explaining the mechanism of departure from hydrogen isotope equilibrium, our model predicts a clear ^2^ε_CH_4_-H_2_O_-ΔG_net_ relation, which has not been accessed by the range of ΔG_net_ explored in laboratory cultures to date.

The hydrogen atom added to CH-H_4_MPT may come from F_420_ (which exchanges hydrogen atoms with H_2_O) in the methylene-H_4_MPT dehydrogenase (Mtd)-catalyzed reaction or from H_2_ in the H_2_-dependent methylene-H_4_MPT dehydrogenase (Hmd)-catalyzed reaction (Fig. 1A) (*53*). Thus, up to one-quarter of the hydrogen atoms in methane may come directly from H_2_, depending on the relative activity of Mtd and Hmd. Intracellular H_2_ rapidly exchanges hydrogen atoms with H_2_O due to the activity of various hydrogenases, and this exchange results in isotopic equilibrium (*54*, *55*) in which H_2_ is strongly depleted in deuterium relative to H_2_O ($\alpha_{H_{2}O‐H_{2}}^{\text{eq}}$ of ≈3 at 60°C) (*56*). High growth rates at high H_2_ concentrations favor Hmd activity over Mtd (*35*, *53*). Under these conditions, although the H_2_ may itself be in hydrogen isotope equilibrium with the H_2_O, kinetically controlled incorporation of hydrogen isotopes directly from D-depleted H_2_ may result in disequilibrium ^2^ε_CH_4_-H_2_O_ (Fig. 3, D and E). In some cases, which probably only occur under extreme experimental conditions such as isotopic labeling, intracellular H_2_O-H_2_ isotopic disequilibrium may exist. If Hmd is active under these conditions, then ^2^ε_CH_4_-H_2_O_ may vary in response to variations in the hydrogen isotope composition of H_2_. Our model captures this behavior, as observed in a rapidly growing methanogenic culture at 60°C (*20*), which displayed ^2^ε_CH_4_-H_2_O_ values between −145 and −480‰ that covary with the H_2_O-H_2_ hydrogen isotope fractionation (Fig. 3F).

### Clumped isotopologue distributions

**Fig. 4.**
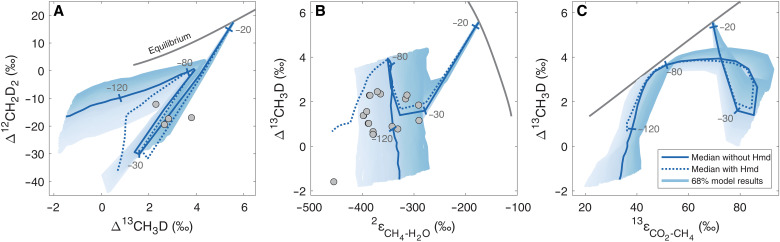
Model-laboratory culture comparison of clumped isotopologue abundances. Experimental data (gray circles) and model results (lines and envelopes) of 200 simulations at 30°C. The dark gray lines represent temperature-dependent isotopic equilibrium at 0° to 350°C, and the thick blue lines show the median of the simulations, with tick marks at ΔG_net_ values of −20, −30, −80, and −120 kJ mol^−1^. The envelopes represent 68% of 200 model simulations, with KFFs drawn from calibrated distributions. (**A**) ∆^13^CH_3_D against ∆^12^CH_2_D_2_. (**B**) ∆^13^CH_3_D against ^2^ε_CH_4_-H_2_O_. (**C**) ∆^13^CH_3_D against ^13^ε_CO_2_-CH_4__. Laboratory culture samples are from hydrogenotrophic methanogens that do not have membrane-associated methanophenazines. The model results under thermophilic conditions are in fig. S9.

The abundances of the doubly substituted isotopologues of methane (^13^CH_3_D and ^12^CH_2_D_2_) are expressed as a deviation from their concentrations at a stochastic distribution of the rare isotopes, with Δ^13^CH_3_D = ^^13^CH_3_^^D^*R*_sample_/^^13^CH_3_^^D^*R*_stochastic_ − 1 [‰] and Δ^12^CH_2_D_2_ = ^^12^CH_2_^^D2^*R*_sample_/^^12^CH_2_^^D2^*R*_stochastic_ − 1 [‰]. Both Δ^13^CH_3_D and Δ^12^CH_2_D_2_ values depend on the methane formation temperature (*57*), yet applications of methane clumped isotopes to constrain its formation temperature and mechanism are complicated by source mixing and disequilibrium effects (*23*, *58*). The dependence of Δ^13^CH_3_D and Δ^12^CH_2_D_2_ on ΔG_net_ has not been determined experimentally, and our full metabolic-isotopic model predicts that departure from clumped isotope equilibrium is nonmonotonic (Fig. 4 and fig. S7), in contrast with previous estimates of these relations that were based on simpler models (*22*, *23*). As ΔG_net_ becomes negative, both ^13^CH_3_D and Δ^12^CH_2_D_2_ values decrease from the expected equilibrium compositions, and Δ^12^CH_2_D_2_ becomes anticlumped (i.e., <0‰) because of expression of the KFFs of the Mcr- and Mtr-catalyzed reactions (fig. S7). After the initial decrease in Δ^13^CH_3_D and Δ^12^CH_2_D_2_, both increase with increasingly negative ΔG_net_, and Δ^13^CH_3_D increases to values almost as high as the equilibrium values (Fig. 4A). This behavior has two implications. First, there is a range of ΔG_net_ (≈−70 to −90 kJ mol^−1^) over which Δ^13^CH_3_D values may give the false appearance of proximity to isotopic equilibrium. Second, there is a range of ΔG_net_ (≈−30 to −80 kJ mol^−1^) over which Δ^13^CH_3_D and Δ^12^CH_2_D_2_ cannot uniquely constrain the energetic state of the cell (e.g., Δ^13^CH_3_D is ≈4‰ at both ΔG_net_ of ≈−25 and ≈−80 kJ mol^−1^). However, in combination with ^13^ε_CO_2_-CH_4__ and ^2^ε_CH_4_-H_2_O_ data, the position in the ΔG_net_ landscape and the degree of departure from equilibrium may be uniquely constrained (Fig. 4, B and C). The multiple-isotope composition of methane (i.e., bulk carbon and hydrogen and clumped isotopes), interpreted within the quantitative framework provided by our metabolic-isotopic model, may thus be a useful proxy for ΔG_net_ in environments where measurements of H_2_, CO_2_, and CH_4_ concentrations in the cells’ immediate vicinity are not easily obtainable. In addition to departure from equilibrium of the Mcr- and Mtr-catalyzed reactions, which may cause anticlumped Δ^12^CH_2_D_2_ compositions, another testable prediction of our model is that the Hmd-catalyzed reaction may also cause anticlumping. This arises from combinatorial effects, which are caused by mixing of hydrogen atom sources with a different hydrogen isotope composition (δD) and are proportional to the difference in δD values between the sources (*59*–*61*). The combinatorial effects are expected to be most clearly expressed under conditions that promote H_2_O-H_2_ hydrogen isotope disequilibrium, such as isotope labeling experiments (fig. S8).

### Apparent versus actual isotopic equilibrium in laboratory cultures

Our predicted trajectories for departure from equilibrium ^13^ε_CO_2_-CH_4__, ^2^ε_CH_4_-H_2_O_, Δ^13^CH_3_D, and Δ^12^CH_2_D_2_ values and comparison to available data suggest that, to date, carbon, hydrogen, and clumped isotope equilibrium has not been obtained in methanogenic laboratory cultures. This is not entirely unexpected, as biological activity is, by definition, out of equilibrium. However, if ΔG_net_ is small enough, isotopic fractionations can be practically indistinguishable from true isotopic equilibrium. In several experiments, near-equilibrium ^13^ε_CO_2_-CH_4__ values co-occur with clearly disequilibrium ^2^ε_CH_4_-H_2_O_ values, which have been previously explained by a decoupling of the carbon and hydrogen isotope systems in methanogenesis (*20*, *62*). On the basis of the results of our mechanistic metabolic-isotopic model, we suggest instead that the measured near-equilibrium ^13^ε_CO_2_-CH_4__ values did not reflect isotopic equilibrium but the descending branch from the ^13^ε_CO_2_-CH_4__ maximum (which occurs at ΔG_net_ ≈−40 kJ mol^−1^) with increasingly negative ΔG_net_. That is, we suggest that apparent equilibrium ^13^ε_CO_2_-CH_4__ values may emerge by a fortuitous combination of EFFs and KFFs and not because of actual isotopic equilibrium between CO_2_ and methane. We further suggest that experiments aimed at constraining isotopic equilibrium should be conducted at ΔG_net_ values that are not more negative than −20 kJ mol^−1^. At more negative values, expression of KFFs will result in disequilibrium isotopic fractionations. Our model links ΔG_net_ values to the specific rates of methanogenesis (Fig. 1 and fig. S10), which are sometimes easier (than ΔG_net_) to quantify in laboratory cultures and natural environments. Thus, our model may serve to plan future experiments in which the rate of methanogenesis is slow enough for sufficient isotopic exchange between methane, CO_2_, and H_2_O to achieve near-equilibrium isotopic distributions and internal equilibration of the clumped isotopologues.

### Isotopic fractionation in energy-limited environments

The metabolic-isotopic model may be used to examine the controls on the isotopic fractionation of methanogenesis not only in laboratory cultures but also in natural environments. Laboratory cultures operate far from equilibrium, usually at [H_2_] and csMRs much higher than those in natural environments (table S6 and fig. S10). Recent reevaluations of slowly forming biogenic methane sources such as marine sediments, coal beds, or shale gas deposits revealed that apparent CH_4_-CO_2_ and CH_4_-H_2_O isotopic equilibrium is common (*20*, *41*, *43*, *63*–*65*). We used our model to study the isotopic effects in these settings by adopting enzyme kinetic parameters obtained from methanogens grown under energy limitation. These conditions promote the activity of the Mcr I isoenzyme at the expense of the Mcr II isoenzyme (see Methods) (*35*, *66*). We used the modified model to calculate the csMR and isotopic effects at 0° to 60°C and assessed the resulting ΔG_net_-csMR-isotopic relations.

Existing estimates of environmental ΔG_net_ of hydrogenotrophic methanogenesis are more positive than −30 kJ mol^−1^ (table S6). However, the determination of ΔG_net_ in natural environments is often difficult because of low and spatially heterogeneous in situ [H_2_] (*67*–*69*), and the actual range of ΔG_net_ likely reflects this heterogeneity. As ΔG_net_ determines the csMR (fig. S10), which is easier to measure, we henceforth discuss csMR-isotopic fractionation relations. Our model predictions of csMR in energy-limited environments (10^−5^ to 1 fmol cell^−1^ day^−1^) are considerably lower than the typical range in laboratory cultures (10 to 10^4^ fmol cell^−1^ day^−1^; fig. S10) (*21*, *52*). This csMR difference of several orders of magnitude reflects the model calibration to reproduce [H_2_]-csMR relations in low-[H_2_] laboratory culture experiments (Fig. 1B). Although this calibration was limited to available experimental [H_2_] higher than 1 μM, our predicted csMR values at about nanomolar H_2_ concentrations (10^−5^ to 1 fmol cell^−1^ day^−1^) overlap with the range of csMR values from shallow and deep marine sediments (10^−4^ to 1 fmol cell^−1^ day^−1^), which we calculated from reported bulk methanogenesis rates and cell densities (table S7). We note that there is large uncertainty regarding these environmental csMR values, which stems, in part, from uncertainty on the net rate of methanogenesis. The methanogenesis rate in natural environments is often determined by radiotracer assays, which may lead to an overestimation of the net methanogenesis rate by orders of magnitude if the reversibility of net methanogenesis (i.e., between CO_2_ and methane) is higher than 0.9 (Fig. 2). An additional source of uncertainty is the number of active methanogenic cells in the sediments, and there are currently very limited estimates of these values. Despite these uncertainties, our model predictions of csMR match not only environmental csMR estimates but also estimates of cell-specific power utilization, *P* = − ΔG_net_ × csMR (in watts per cell). We predict *P* between 10^−22^ and 10^−18^ W cell^−1^ for ΔG_net_ between −10 to −30 kJ mol^−1^, respectively, at 10°C, in agreement with previously estimated cell-specific power utilization in marine sediments (*70*).

**Fig. 5.**
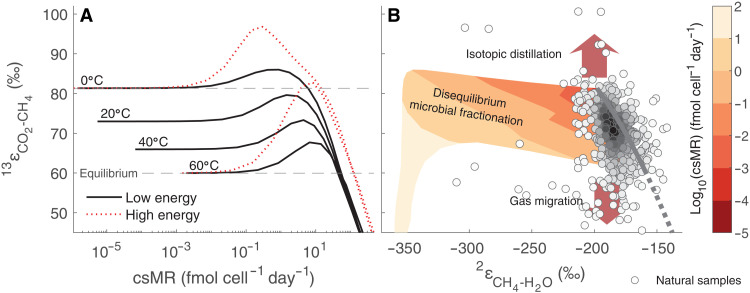
Isotopic fractionation during methanogenesis under energy-limited conditions. (**A**) ^13^ε_CO_2_-CH_4__ against csMR. Dotted red lines show the laboratory-calibrated model (high-energy) results at 0° and 60°C. (**B**) ^13^ε_CO_2_-CH_4__ against ^2^ε_CH_4_-H_2_O_. Contours are log_10_(csMR) predicted by our model, between 0° and 60°C. The calculations are for [H_2_] between 1 nM and 5 μM, [CO_2_] and [CH_4_] of 1 mM, and a cell volume of 1 μm^3^. The circles are biogenic environmental samples from marine sediments, coal bed methane, and natural gas deposits (*n* = 491), colored by the frequency of occurrence and showing that most samples plot close to the isotopic equilibrium curve.

The model reveals that in contrast to methanogenesis in lab cultures, under energy-limited conditions, the Mtr-catalyzed reaction departs from equilibrium before the Mcr-catalyzed reaction (fig. S11). As a consequence, different csMR-fractionation relations emerge, most notably, an initially attenuated response of ^13^ε_CO_2_-CH_4__ values to increasing csMR (Fig. 5A). Specifically, ^13^ε_CO_2_-CH_4__ displays a much more muted increase to larger-than-equilibrium values with increasing csMR in the low energy–calibrated model than in the high energy–calibrated model. The reason for this apparent carbon isotopic equilibrium is the smaller magnitude of the KFF of Mtr relative to Mcr (−28 and −38‰ at 60°C, respectively; fig. S3). Thus, as the Mtr-catalyzed reaction departs from equilibrium, the net carbon isotope fractionation remains within ≈5‰ of ε13CO2‐CH4eq up to a csMR of ∼10 fmol cell^−1^ day^−1^ (Fig. 5). An environmental prediction of the above is that ^13^ε_CO_2_-CH_4__ that differs measurably from ε13CO2‐CH4eq indicates csMR higher than ∼10 fmol cell^−1^ day^−1^.

Analytically distinguishable departure of ^2^ε_CH_4_-H_2_O_ from equilibrium occurs at csMR between ∼0.001 and 0.1 fmol cell^−1^ day^−1^ (at 0° and 60°C, respectively; fig. S12B), and departure from clumped isotope equilibrium occurs over a similar range of csMR (fig. S12, C and D). If hydrogenotrophic methanogens can be cultured at such low csMR (small negative ΔG_net_), then an implication of the model csMR-isotopic fractionation relations is that experimental studies aimed at measuring methane that is close to bulk hydrogen isotope equilibrium would accumulate enough methane for analysis within a few days. In contrast, given existing analytical capabilities, accumulation of enough methane for clumped isotope analysis at csMR low enough to achieve isotopic near equilibrium requires combinations of long experimental duration (hundreds of days) and large culture volume (hundreds of milliliters).

In addition to its utility for the design of culture experiments, our model predicts that at csMR between 0.1 and 10 fmol cell^−1^ day^−1^ , one should expect near-equilibrium ^13^ε_CO_2_-CH_4__ concurrent with disequilibrium ^2^ε_CH_4_-H_2_O_. The opposite situation (i.e., near-equilibrium ^2^ε_CH_4_-H_2_O_ and disequilibrium ^13^ε_CO_2_-CH_4__) is common in natural environments, although csMR is usually unknown. As suggested in previous studies, this apparent hydrogen isotope equilibrium concurrent with carbon isotope disequilibrium may be explained by diffusive mixing of CO_2_ and/or methane from different depths within the sediment or isotopic (Rayleigh) distillation (Fig. 5B) (*41*, *71*, *72*). Rayleigh distillation is not expected to be relevant for H_2_O, as its pool is large enough so that its composition will not change under environmentally plausible conditions. This is not the case for the much smaller dissolved inorganic carbon pool, which can evolve isotopically upon progressive consumption. If disequilibrium ^13^ε_CO_2_ -CH_4__ is explained as above (transport and/or Rayleigh distillation) rather than by microbial expression of KFFs, then the scarcity of data within the field representing disequilibrium microbial fractionation in Fig. 5B may reflect near-equilibrium isotopic fractionation during methanogenesis in energy-limited environments. An alternative explanation, in which methane originally forms in both ^13^ε_CO_2_-CH_4__ and ^2^ε_CH_4_-H_2_O_ disequilibrium (e.g., csMR > 10 fmol cell^−1^ day^−1^ according to our model; Fig. 5A) and subsequent enzymatically catalyzed hydrogen isotope exchange promotes ^2^ε_CH_4_-H_2_O_ equilibrium (*20*), may not be consistent with the landscape of departure from equilibrium of the individual reactions predicted by our model. In this alternative explanation, diffusion of methane in and out of the cells and enzymatically catalyzed hydrogen isotope exchange in the last steps of the methanogenesis pathway (the Mcr- and Hdr-catalyzed reactions) without net methane production, and possibly much later than the timing of methane formation, are suggested to lead to ^2^ε_CH_4_-H_2_O_ equilibrium, while disequilibrium upstream of these reactions sustains ^13^ε_CO_2_-CH_4__ disequilibrium. However, we note that the Mcr- and Hdr-catalyzed reactions are the first to depart from equilibrium (Fig. 1 and fig. S11). Our model reveals that near equilibrium for these reactions, which is required to approach CH_4_-H_2_O hydrogen isotope equilibrium, implies near equilibrium in the entire methanogenesis pathway. In this case, carbon isotope equilibration is also expected, contrary to the observations, suggesting that postformation hydrogen isotope equilibration is not the cause for apparent equilibrium^2^ε_CH_4_-H_2_O_.

If, as argued above, apparent equilibrium ^2^ε_CH_4_-H_2_O_ in natural environments is the outcome of near-equilibrium isotopic fractionation during methanogenesis, and not disequilibrium microbial fractionation followed by Mcr- and Hdr-catalyzed equilibration of hydrogen isotopes, then implied csMR is lower than ∼0.001 to 0.1 fmol cell^−1^ day^−1^ (depending on temperature). This predicted range of csMR is consistent with almost all csMR estimated from cell abundances and volumetric methanogenesis rates (table S7). In environments that support higher csMR and disequilibrium microbial fractionation, near-equilibrium ^2^ε_CH_4_-H_2_O_ concurrent with disequilibrium ^13^ε_CO_2_-CH_4__ may instead be explained by methane cycling or anaerobic oxidation of methane that operates close to the thermodynamic limit of this metabolism (*73*). Alternatively, energy- and substrate-limited methanogenesis from methylated substrates (e.g., acetate and methanol) is expected to generate methane with more positive δ^13^C values (*74*) and, accordingly, smaller ^13^ε_CO_2_-CH_4__ values that depart from CO_2_-CH_4_ carbon isotope equilibrium.

By accounting for the metabolites and reactions in the hydrogenotrophic methanogenesis pathway, we link environmental substrate and product concentrations, pH, and temperature to the energetics and net rate of methanogenesis and to the associated fractionation of carbon, hydrogen, and clumped isotopes. The landscape of departure of individual reactions in the pathway from reversibility controls these fractionations, mechanistically explaining rate-fractionation relations in laboratory cultures. We suggest that a combination of ^13^ε_CO_2_-CH_4__, ^2^ε_CH_4_-H_2_O_, ∆^13^CH_3_D, and ∆^12^CH_2_D_2_ data, interpreted within a metabolic-isotopic model framework, can uniquely constrain the in situ energetics of methanogenic activity (i.e., the actual ΔG_net_ of methanogenesis), which may be difficult to constrain by other means. However, application of such an approach is limited to natural and artificial environments in which hydrogenotrophic methane production is the dominant control over the apparent CH_4_-CO_2_ and CH_4_-H_2_O isotopic fractionations and where other processes are less important. These processes include CO_2_ and methane diffusion, isotopic distillation, and methane cycling, which are prevalent in natural environments. Nevertheless, the mechanistic understanding of isotopic effects during microbial methanogenesis provided by our model allows an inference that this metabolism operates close to chemical and isotopic equilibrium in a wide range of natural environments, including marine sediments, coal beds, and natural gas deposits.

## METHODS

### Relating net isotopic fractionation to thermodynamic drive

The metabolic-isotopic model that we developed is based on a general framework, previously used to investigate sulfur isotopes in microbial sulfate reduction (*25*, *38*). This approach is applicable to any metabolic network with some modifications that we present below. In general, the net flux of a reversible enzymatically catalyzed reaction (*J*) is defined by the difference between the forward (*J*^+^) and backward (*J*^−^) gross fluxes and can be expressed as (*26*)

$$\begin{array}{c} J=J^{+}-J^{-}=V^{+}\times (\frac{\prod_{j}{\left([r_{j}]/K_{M_{j}}\right)}^{n_{j}}}{1+\prod_{j}{\left([r_{j}]/K_{M_{j}}\right)}^{n_{j}}+\prod_{i}{\left([p_{i}]/K_{M_{i}}\right)}^{m_{i}}})\\ \times (1-e^{\Delta G_{r}/\mathrm{RT}}) \end{array}$$ (3)

where *V*^+^ is the maximal rate capacity of the enzyme (defined by $[E]\times k_{\text{cat}}^{+}$, where $k_{\text{cat}}^{+}$ is the catalytic rate constant for the forward reaction and [*E*] is the enzyme concentration), [*r_j_*] and [*p_i_*] are concentrations of the *j*th reactant and *i*th product, respectively, and *K*_M*_j_*_ and *K*_M*_i_*_ are the Michaelis constants for the forward and reverse directions, respectively. The *n_j_* and *m_i_* stand for the stoichiometry of the reactant and product, respectively. A net flux, as described by Eq. 3, is calculated for each of the reactions in the hydrogenotrophic methanogenesis pathway (table S1). These fluxes are used to construct a mass balance, which is solved at a steady state to find the concentrations of the metabolites. The steady-state solution was obtained by forward integration using ode15s, an ordinary differential equation (ODE) solver in MATLAB. Metabolite concentrations in the model span six to seven orders of magnitude, but all are linked by identical net rates, and this results in orders of magnitude differences among the residence times of the different metabolites. The set of ODEs is thus “stiff,” requiring a solution algorithm that would prevent unreasonably small time steps, which could lead to long computation times or failure of the model. This problem thus required the use of the ode15s solver with an absolute tolerance of 10^−20^ M. We checked that the duration of integration was sufficient to reach a steady-state solution (i.e., no change in concentrations and fluxes) in all cases.

The model’s inputs include the environmental temperature, pH, and extracellular aqueous concentrations of H_2_, CO_2_, and CH_4_. We assume that the intracellular concentrations of H_2_ and CH_4_ are equal to the extracellular concentrations due to their rapid diffusion through the membrane. For CO_2_, we apply a simple diffusion model to relate intracellular to extracellular CO_2_ concentrations. The model’s tunable parameters include enzyme kinetic constants (*K*_M_ and *V*^+^), thermodynamic constants ($\Delta {G^{\prime }}_{r}^{0}$), and cellular parameters such as cell size and concentrations of some of the metabolites. We elaborate on the parameters and the choice of their values below. With the inputs prescribed and values for the tunable parameters chosen, the model outputs are the concentrations of all intracellular metabolites and the gross fluxes among these metabolites, which are related to the reactions in the methanogenesis pathway.

We used the forward and backward gross fluxes from the metabolic model to calculate the net isotopic fractionations in hydrogenotrophic methanogenesis. To this end, we constructed a mass balance for each isotopic system (for both bulk and clumped isotopes), which is based on calculating an isotopic flux associated with each of the chemical fluxes. For the schematic reaction *r* → *p*, this isotopic flux can be approximated

$$F_{\mathrm{rp}}=J_{\mathrm{rp}}^{+}R_{r}\alpha_{\mathrm{rp},r}$$ (4)

where $J_{\mathrm{rp}}^{+}$ is the forward chemical flux between metabolite pools *r* and *p*, *R_r_* is the abundance ratio of heavy to light isotopes in pool *r*, and α_*rp*,*r*_ is the KFF of the reaction. A term similar to Eq. 4 was assigned to account for the consumption and production of each (singly and doubly substituted) isotopologue in the pathway while considering the stoichiometry and the symmetry coefficients, where relevant, in a similar manner to previous model derivations (*23*, *24*). As described above, these terms were used to construct isotopic mass balances in the form of a set of coupled differential equations, which were solved numerically using the ode15s solver in MATLAB to obtain the steady-state bulk and clumped isotopic composition of all intracellular metabolites, given bulk extracellular D/H and ^13^C/^12^C of H_2_O and CO_2_, respectively.

The net isotopic fractionation in linear metabolic networks such as the carbon reaction network in hydrogenotrophic methanogenesis can be solved analytically. For example, in the case of the reaction *r* ⇄ *p*, the net isotopic fractionation between the metabolite pools of *r* and *p* ($\alpha_{r,p}^{\text{net}}$) can be calculated by

$$\alpha_{r,p}^{\text{net}}=(\alpha_{r,p}^{\text{eq}}-\alpha_{r,\mathrm{rp}})\times (J_{\mathrm{pr}}^{-}/J_{\mathrm{rp}}^{+})+\alpha_{r,\mathrm{rp}}$$ (5)

where $\alpha_{r,p}^{\text{eq}}$ is the EFF between pools *r* and *p* and $\alpha_{r,\mathrm{rp}}$ is 1/KFF of the forward reaction. A full derivation for this term is presented in a previous study (*25*). The reversibility $J_{\mathrm{pr}}^{-}/J_{\mathrm{rp}}^{+}$ is directly related to $\Delta G_{r,i}^{\prime }$ through the flux-force relationship (Eq. 2).

Equation 5 can be expanded recursively to describe the net isotopic fractionation in a linear reaction network of arbitrary length (*25*). Such a recursive expression has the advantage of a significantly reduced computational time compared to the numerical solution that is used for nonlinear reaction networks. We verified that this analytical solution yields identical results to the numerical solution for the net carbon isotopic fractionation. We note that the solution in Eq. 5 is irrelevant for branched networks, such as those describing methane hydrogen isotopes or clumped isotopologues, and that an analytical solution for the full ODE sets of these systems is not feasible. Hydrogen and clumped isotopes require the computationally heavier numerical simulations that are described above.

### Metabolic model parameters

We tested the model for input [H_2_] ranges of 10^−9^ to 10^−2^ M and [CO_2_] ranges of 0.1 to 100 mM, and [CH_4_] was held at 10^−5^ M. These ranges were chosen to include concentrations reported in various culture experiments and guided by a sensitivity analysis, which showed that the model is relatively insensitive to [CH_4_] but that it is sensitive to [H_2_] and [CO_2_]. In the figures, we present the model results for [CO_2_] of 10 mM, [CH_4_] of 10 μM, and at 60°C, unless stated otherwise. We numerically solve for the steady-state concentrations of 16 metabolites in the hydrogenotrophic methanogenesis pathway of *Methanothermobacter thermoautotrophicus*. The initial concentrations of the total (i.e., reduced and oxidized) electron carriers (Fd, HS-CoB, and F_420_) and H_4_MPT were compiled from experimentally determined concentrations, where available (table S8).

### Extracellular and intracellular concentrations of CO_2_, H_2_, and CH_4_

The model was designed to predict intracellular metabolite concentrations of the hydrogenotrophic methanogenesis pathway at a steady state by prescribing temperature and extracellular [H_2_], [CO_2_], and [CH_4_]. These small, nonpolar molecules diffuse rapidly through the cell membrane, and it is often assumed that their intracellular aqueous concentrations are in equilibrium with the headspace gas (related to the aqueous concentrations through Henry’s law constants; table S8). We adopt this assumption for CH_4_, to which the model is relatively insensitive. However, we explore two cases in which concentrations of CO_2_ and H_2_ are not in equilibrium with the headspace: (i) CO_2_ diffusion through the membrane may become limiting with increasing net methanogenesis flux (*75*). We model intracellular CO_2_ concentrations ([CO_2(in)_]) using Fick’s law

$$J_{\text{dif}}=D\times A\times \frac{\left[CO_{2(\text{out})}]-[CO_{2(\text{in})}\right]}{z}$$ (6)

where *J*_dif_ is the net CO_2_ diffusive flux, *D* is the permeability constant, *A* is the membrane surface area, *z* is the membrane width, and [CO_2(out)_] is the extracellular CO_2_ concentration. To determine *A*, we assume that the cells are spherical and relate their volume (*V*_cell_) to *A* via the cell’s radius (*r*_cell_), where

$$r_{\text{cell}}=\sqrt[{3}]{\frac{3}{4}\frac{V_{\text{cell}}}{\pi }}$$ (7)

and

$$A=4\times \pi \times r_{\text{cell}}^{2}$$ (8)

The values that we used for *D*, *z*, and *V*_cell_ are listed in table S8. (ii) Cultivation of methanogens under low [H_2_] conditions requires growth in cocultures with H_2_-producing (hydrogenic) bacteria (*17*, *20*). Juxtaposition of methanogens and hydrogenic bacteria may result in immediate consumption of bacterially produced H_2_ by the methanogens, which may result in spatially heterogeneous H_2_ concentrations (*67*, *68*). In this case, the aqueous H_2_ concentration in the immediate vicinity of the methanogens may be higher than the H_2_ concentration in equilibrium with the headspace. The best fit between the net carbon isotopic fractionation in methanogenesis (^13^ε_CO_2_ − CH_4__) measured in laboratory cocultures and predicted in our model is obtained at in situ H_2_ concentrations that are approximately five times higher than the concentration at equilibrium with the headspace.

### Energy conservation

Methanogens conserve chemical energy by coupling the exergonic methyl group transfer by the Mtr-catalyzed reaction to export of Na^+^ ions out of the cell

$$\begin{array}{c} {\text{CH}}_{3}‐H_{4}\text{MPT}+\text{HS-CoM}+x{\text{Na}}_{\text{in}}^{+}⇄\\ {\text{CH}}_{3}‐\text{SCoM}+H_{4}\text{MPT}+x{\text{Na}}_{\text{out}}^{+} \end{array}$$ (9)

where *x* is the stoichiometric coefficient for Na^+^ translocation per cycle of the reaction. Na^+^ translocation generates an electrochemical gradient, which is used to phosphorylate adenosine diphosphate to ATP by a proton- or sodium-driven adenosine triphosphatase (*76*). Hydrogenotrophic organisms without membrane-associated methanophenazines, such as *M. thermoautotrophicus*, have an ATP yield (*Y*_ATP_) of 0.5 mol of ATP per mol of methane under optimal growth conditions (*46*). The standard transformed Gibbs free energy of ATP production under physiological conditions (ΔG′^m^_r, ATP_) is +43.5 kJ (*77*), and thus, 0.5 moles of ATP require an electrochemical gradient of at least −22 kJ. We assume that the cells use the minimal electrochemical potential possible, so the *x* in Eq. 9 is equivalent to *Y*_ATP_. On the basis of the observations that the minimal threshold for methanogenic activity is ≈−10 kJ mol^−1^ (*46*), we estimate that under nonoptimal growth conditions, *Y*_ATP_ is closer to 0.2, and we thus use a default *Y*_ATP_ value of 0.2. The value of *Y*_ATP_ affects the reversibility of the Mtr-catalyzed reaction, which is calculated by

$$\begin{array}{c} 1-\frac{\left[{\text{CH}}_{3}‐\text{SCoM}][H_{4}\text{MPT}\right]}{\left[{\text{CH}}_{3}‐H_{4}\text{MPT}][\text{HS-CoM}\right]}\\ \times \text{exp}(\frac{\Delta G_{r,\text{Mtr}}^{\prime 0}+Y_{\text{ATP}}\times \Delta G_{r,\text{ATP}}^{\prime m}}{\mathrm{RT}}) \end{array}$$ (10)

Thus, the choice of *Y*_ATP_ controls the relative expression of the KFF and EFF of the Mtr-catalyzed reaction and, consequently, the net fractionation of the entire pathway. The choice of *Y*_ATP_ value also sets the smallest ΔG_net_ required for methanogenic activity, which reflects the minimal energetic requirement to produce ATP. For example, with the choice of *Y*_ATP_ = 0.2, there is model methanogenic activity only at ΔG_net_ < ≈−10 kJ mol^−1^. This means that ^13^ε_CO_2_-CH_4__, ^2^ε_CH_4_-H_2_O_, and clumped isotopologue distributions cannot attain combinations of values typical of isotopic near equilibrium (ΔG_net_ between −10 and 0 kJ mol^−1^). We explore the sensitivity of the model to *Y*_ATP_ in figs. S13 to S15. The effect is most noticeable on ^13^ε_CO_2_-CH_4__, where a smaller *Y*_ATP_ value (0.1) induces an earlier departure of the Mtr-catalyzed reaction from equilibrium, which results in a smaller peak in ^13^ε_CO_2_-CH_4__ values relative to the default *Y*_ATP_ value of 0.2. A larger *Y*_ATP_ value (0.3) induces a later departure of the Mtr-catalyzed reaction from equilibrium, which causes a larger peak to larger-than-equilibrium ^13^ε_CO_2_-CH_4__ values. A best fit between the model and culture experiments is obtained with *Y*_ATP_ of 0.2.

### Enzyme kinetic parameters

The enzyme *K*_M_ values of 10 metabolites were absent in the literature. On the basis of the observation that *K*_M_ values in various organisms are normally distributed (*78*), we generated distributions for the missing *K*_M_ values. Wherever possible, we determined the mean and SDs of these prior distributions based on metabolite-specific *K*_M_ values from other enzymes deposited in the BRENDA database (*79*). We used the model-measurement mismatch to produce posterior distributions for these *K*_M_ values, with the following procedure: (i) Run the metabolic model 10^4^ times, drawing the missing parameters from prior distributions (table S5). (ii) Weigh the *K*_M_ value drawn from the prior distribution by the model-experimental mismatch of the biomass-specific methanogenesis rate, which were measured for cells grown under optimal conditions in varying H_2_ concentrations (*66*). The weight was defined as the inverse of the squared sum of squared errors (1/SSE^2^) (fig. S2). (iii) Run the metabolic model with the weighted posterior distributions. In the isotopic model, we used the median *K*_M_ values from the posterior distributions, which we refer to in the various figures as “calibrated distributions” (table S4).

The maximal rate capacity *V*^+^ (Eq. 3) is a product of an enzyme’s turnover rates ($k_{\text{cat}}^{+}$) and its concentration ([*E*]). Enzyme levels are mostly unknown, and to circumvent this, we used rate capacities determined in vitro in pure culture crude cell extracts ($V_{\text{vit}}^{+}$), which provide approximate values of $k_{\text{cat}}^{+}\times$ [*E*] of all enzymes in the pathway (we explore the sensitivity to these values in figs. S13 to S15). There were no *V*^+^ values that were completely missing from the literature. Although this approach avoids the need to prescribe [*E*] and $k_{\text{cat}}^{+}$ values, $V_{\text{vit}}^{+}$ does not necessarily reflect in vivo rate capacities ($V_{\text{viv}}^{+}$). To account for the difference between in vitro and in vivo conditions, we used a scaling factor

$$U_{\text{viv}/\text{vit}}=\frac{{\left[E\right]}_{\text{viv}}\times k_{\text{cat},\text{viv}}^{+}}{{\left[E\right]}_{\text{vit}}\times k_{\text{cat},\text{vit}}^{+}}$$ (11)

Assuming that $k_{\text{cat},\text{vit}}^{+}\approx k_{\text{cat},\text{viv}}^{+}$, this term reduces to *U*_viv/vit_ = [*E*]_viv_/[*E*]_vit_, which implies that this scaling factor is essentially a ratio of the active enzyme concentrations of in vitro and in vivo conditions. We also assumed that this ratio is uniform across all enzymes. To determine *U*_viv/vit_, we used the model-experimental mismatch, as was described for the *K*_M_ values (fig. S2). We found that using a *U*_viv/vit_ value of ≈11 yields the best match between the model and experimental results of biomass-specific methanogenesis rates.

### Temperature effects on methanogenesis rates

The *V*^+^ values in the model were obtained from crude cell extracts that were mostly cultured at 60°C, and we have only limited information to determine the dependence of *V*^+^ values on temperature. In general, temperature is expected to affect the activity of individual enzymes, the thermodynamics of the individual reactions, and likely other whole-organism properties. The activity of single enzymes has well-described temperature dependences, with a typical peak due to changes in the heat capacity of the enzyme (*80*, *81*). The effect of temperature on the net rate of individual reactions in the pathway is built into our metabolic model through the thermodynamic term in Eq. 3 (1 − *e*^ΔG*_i_*/*RT*^). Uncertainty in the temperature dependence of $\Delta {G^{\prime }}_{r}^{0}$ values that have not been determined experimentally currently precludes incorporation of this temperature dependence of net rates in the model.

The effect of temperature on whole-organism activity is more difficult to evaluate, as organisms adapt to maximize activity at different temperatures by a suite of physiological changes, for example, by controlling enzyme expression rates, the spatial density of membrane transporters, and the composition of membranes, with effects on membrane permeability. We account for the coupled dependence of enzyme activity and whole-organism adaptations by assigning an Arrhenius-type temperature dependence term ($Q_{10}^{V^{+}}$) that determines the *V*^+^ for a given temperature

$$V_{2}^{+}=V_{1}^{+}\times {\left(Q_{10}^{V^{+}}\right)}^{\Delta T/10}$$ (12)

where Δ*T* is the temperature difference. We reemphasize that, currently, there are no estimates of the actual magnitude of $Q_{10}^{V^{+}}$. We found that when $Q_{10}^{V^{+}}$ is assigned a value of unity (i.e., csMR increases with temperature only because of temperature effects on the thermodynamic drive), csMR increases by up to three orders of magnitude between 0° and 60°C when ΔG_net_ is a small negative number (≈−15 kJ mol^−1^). This is equivalent to an increase in csMR by a factor of ≈3.2 per 10°C warming. At large negative ΔG_net_, csMR is almost independent of temperature, as the thermodynamic drive is close to unity irrespective of temperature (fig. S10, solid lines). A $Q_{10}^{V^{+}}$ value greater than unity results in a higher temperature sensitivity of csMR. For example, assigning a $Q_{10}^{V^{+}}$ value of 1.5 generates csMR that is ∼4 orders of magnitude lower at 0°C than at 60°C when ΔG_net_ is a small negative value (equivalent to a factor of ≈4.6 increase per 10°C warming) and approximately 10-fold lower when ΔG_net_ has a large negative value (fig. S10, dotted lines). In the model, we used a default value for $Q_{10}^{V^{+}}$ of 1, resulting in a “whole-organism” increase in csMR by a factor of ≈3.2 for every 10°C warming. We note that choosing a $Q_{10}^{V^{+}}$ of 1 does not imply that the activity of single enzymes is independent of temperature but rather that the maximal rate capacity of enzymatically catalyzed reactions may be approximately invariant across temperatures in organisms that are adapted to those temperatures.

### Isotopic model parameters

The temperature-dependent isotope EFFs that are associated with each of the steps in the hydrogenotrophic methanogenesis pathway were calculated using density functional theory at the M06-L/def2-TZVP level of theory with the SMD implicit solvation model (*43*, *82*). The KFFs were determined experimentally only for Mcr (*42*). To account for missing KFFs, we randomly sampled their values from prior uniform distributions 10^6^ times. We included both primary and secondary isotopic effects ($\alpha_{p}^{+}$ and $\alpha_{s}^{+}$, respectively). Primary isotopic effects are due to the breaking or formation of a bond directly with the atom of interest, and secondary isotopic effects are due to breaking or formation of an adjacent bond. Secondary KFFs are usually smaller than primary KFFs. We weighted the combinations of KFFs drawn from the prior distributions by the model-experimental mismatch, expressed as the inverse of the squared sum of squared errors (1/SSE^2^), to generate posterior distributions of the KFFs, which were then used in the model, in a procedure similar to that used to determine the missing *K*_M_ and *U*_viv/vit_ values. We assumed that KFFs are similar between the different temperatures, as we found that separately calibrating the model at each temperature yielded very similar posterior distributions. The reverse KFF for each simulation is calculated from the forward KFF and the temperature-dependent EFF, as required by relation α^eq^ = α^−^/α^+^. This yields slightly different posterior distributions of the reverse KFFs between the two temperatures (fig. S16). We assumed normal KFFs (i.e., the light isotopologues react faster than the heavy isotopologues; α < 1) for both carbon and hydrogen isotopes and assigned $\alpha_{p}^{+}\in (0.4,1)$ [$\alpha_{p}^{+}\in (0.2,1)$ for the Mvh/Hdr-catalyzed reaction] and $\alpha_{s}^{+}\in (0.6,1)$ for hydrogen isotopes and α^+^ ∈ (0.935,1) for carbon isotopes. Notably, optimal model-measurement fits were obtained with an inverse KFF (i.e., α^+^ > 1) for the Mvh/Hdr-catalyzed back reaction. While normal KFFs are more common, in some cases, inverse KFFs emerge, often in a multistep enzymatic reaction that is treated as one composite reaction. In these reactions, chemical and isotopic equilibrium between reaction intermediates before the rate-determining steps may lead to an overall (apparent) inverse KFF, even if the rate-determining step itself has a normal KFF (*83*, *84*). The KFFs of Mcr were assigned distributions based on the experimental measurements (*42*). The carbon KFF was drawn from a normal distribution with a mean of 0.9615 and an SD of 0.01, and the hydrogen KFFs were drawn from normal distributions (for $\alpha_{p}^{+}$ with a mean of 0.41 and an SD of 0.04 and for $\alpha_{s}^{+}$ with a mean of 0.85 and an SD of 0.035).

The posterior distributions of the KFFs (fig. S3) serve as a sensitivity analysis of the model. Posterior distributions that are similar to the prior distributions indicate insensitivity to the model parameter, in this case, the KFF value. For carbon isotopes, the model shows sensitivity to the KFFs of Fmd, Ftr, and Mtr with smallest-mismatch values of −27, −32, and −28‰ at mesophilic conditions, respectively. This sensitivity is in line with our observations of the reactions that determine the trajectory of ^13^ε_CO_2_-CH_4__ departure from equilibrium. For hydrogen isotopes, the model shows sensitivity to most of the primary KFFs and to the secondary KFFs of Fmd and Mtr. Notably, the primary KFFs of Mtd and Frh tend to be small (i.e., closer to unity), with median values between ≈–300 and ≈–260‰.

The KFFs of the clumped isotopologues are expressed as deviations from the product of the KFFs of the singly substituted isotopologues and are denoted by ^*x*, *y*^γ, where *x* and *y* are the rare isotopes and ^*x*, *y*^γ=^*x*, *y*^α^+^/(*^x^*α^+^×*^y^*α^+^). The γ values are typically small and close to unity (*23*). We drew primary and secondary γ values (γ*_p_* and γ*_s_*, respectively) from uniform distributions: ^13,2^γ*_p_* ∈ (0.997,1) and ^13,2^γ*_s_* ∈ (1,1) for ^13^C-D clumping and ^2,2^γ*_p_* ∈ (0.994,1) and ^2,2^γ*_s_* ∈ (1,1) for double D clumping, which are similar to ranges that were explored in previous works (*24*).

### Simulating methanogenesis in energy-limited conditions

The metabolic parameters that we used to explore isotopic fractionation in methanogenesis were curated from cell cultures grown under optimal conditions. However, methanogens tightly regulate gene expression under energy-limiting conditions, resulting in shifts in enzyme specific activities [e.g., (*35*, *53*, *66*, *85*, *86*)]. Specifically, prolonged H_2_ limitation promotes higher activities of Frh, Mvh/Hdr, and the Mcr I isoenzyme, in parallel to a decrease in the activity of Mcr II (*35*, *66*). Mcr I has a higher affinity to the substrates CH_3_-SCoM and HS-CoB relative to Mcr II, allowing the cells to increase the cell-specific respiration rate when H_2_ becomes limiting (*35*, *87*, *88*). Therefore, in the simulations of methanogenesis in natural, energy-limited environments, we used enzyme activities measured in crude extracts from cells that were grown under conditions favoring Mcr I activity (*66*) with *K*_M_ values of Mcr I (table S1). In laboratory cultures, cells under energy limitation decrease their volume in a matter of days or weeks, and a similar phenomenon was observed when comparing cell communities in energy-replete versus energy-deprived environmental settings (*89*). Thus, we use a cellular volume smaller by a factor of two from the default value (2 fl cell^−1^ versus 1 fl cell^−1^).

## Supplementary Materials

### This PDF file includes:

Supplementary Materials Figs. S1 to S16 Tables S1 to S8 References

## Notes

View/request a protocol for this paper from *Bio-protocol*.
