# Supplementary material for: Controls on the isotopic composition of microbial methane
Source: Sci Adv. 2022 Apr 6;8(14):eabm5713. doi: 10.1126/sciadv.abm5713 (PMC8985922; doi:10.1126/sciadv.abm5713)
Supplement: Supplementary file 1 — Supplementary Materials Figs. S1 to S16 Tables S1 to S8 References [file sciadv.abm5713_sm.pdf]

Science Advances
AAAS

Supplementary Materials for

Controls on the isotopic composition of microbial methane

Jonathan Gropp*, Qusheng Jin, Itay Halevy

*Corresponding author. Email: jonathan.gropp@weizmann.ac.il

Published 6 April 2022, *Sci. Adv.* **8**, eabm5713 (2022)
DOI: 10.1126/sciadv.abm5713

The PDF file includes:

Supplementary Materials
Figs. S1 to S16
Tables S1 to S8
References

Other Supplementary Materials for this manuscript include the following:

Tables S9 and S10
Data S1

Supplementary Materials

Carbon isotope fractionation between CO_2 and a biomass precursor

The carbon isotope composition of biomass of the cell's biomass ($\delta^{13}C_B$) can be described by the isotopic mass balance:

$$\delta^{13}C_B = \frac{1}{3}\left(\delta^{13}C_{CO_2} - {}^{13}\varepsilon_{CO_2\text{-}CH_3} - {}^{13}\varepsilon_{CH_3\text{-}CH_3(Ac)}\right) + \frac{1}{3}\left(\delta^{13}C_{CO_2} - {}^{13}\varepsilon_{CO_2\text{-}CO}\right) + \frac{1}{3}\left(\delta^{13}C_{CO_2} - {}^{13}\varepsilon_{CO_2\text{-}COO}\right)\ , \tag{S1}$$

where $\delta^{13}C_{CO_2}$ is the carbon isotope composition of CO_2, and the ε terms are the net carbon isotope fractionations between CO_2 and CH_3-H_4MPT (${}^{13}\varepsilon_{CO_2\text{-}CH_3}$), between CH_3-H_4MPT and the methyl moiety in acetyl-CoA (${}^{13}\varepsilon_{CH_3\text{-}CH_3(Ac)}$), between CO_2 and the carbonyl moiety in acetyl-CoA (${}^{13}\varepsilon_{CO_2\text{-}CO}$), and between CO_2 and the additional (third) carboxyl group added to acetyl-CoA to form pyruvate (${}^{13}\varepsilon_{CO_2\text{-}COO}$). We found a relatively constant offset of $\approx$20$\pm$10‰ between model ${}^{13}\varepsilon_{CO_2\text{-}CH_3}$ and observed CO_2-biomass carbon isotope fractionations (${}^{13}\varepsilon_{CO_2\text{-}B}$) at a given ${}^{13}\varepsilon_{CO_2\text{-}CH_4}$(fig. S6), which implies that:

$$\delta^{13}C_B \approx \delta^{13}C_{CO_2} - {}^{13}\varepsilon_{CO_2\text{-}CH_3} - 20‰\ . \tag{S2}$$

Combining Eqns. S1 and S2 yields:

$${}^{13}\varepsilon_{CO_2\text{-}CO} + {}^{13}\varepsilon_{CO_2\text{-}COO} + {}^{13}\varepsilon_{CH_3\text{-}CH_3(Ac)} \approx 2 \times {}^{13}\varepsilon_{CO_2\text{-}CH_3} + 60‰\ . \tag{S3}$$

If, for example, ${}^{13}\varepsilon_{CO_2\text{-}CH_3}$ is 30‰, as in the extreme right of fig. S6, the sum of the three fractionations on the left side of Eq. S3 is 120‰, while if ${}^{13}\varepsilon_{CO_2\text{-}CH_3}$ is -20‰, as in the extreme left of fig. S6, the sum of the three fractionations is 0‰.

Sensitivity analysis

We conducted a sensitivity analysis to the tunable model parameters by examining the effect of a 3-fold increase or decrease in the value of these parameters on the reversibility of the enzymatically-catalyzed reactions (fig. S13) and on ${}^{13}\varepsilon_{CO_2\text{-}CH_4}$ (S14) and ${}^{2}\varepsilon_{CH_4\text{-}H_2O}$ (S15). We find that the values

of $^{13}\varepsilon_{CO_2\text{-}CH_4}$ and $^{2}\varepsilon_{CH_4\text{-}H_2O}$ are sensitive only to some of the model parameters, but that the overall trajectories of the isotopic fractionation dependence on ΔG_{net} are preserved. We identified three types of sensitivity of $^{13}\varepsilon_{CO_2\text{-}CH_4}$ and $^{2}\varepsilon_{CH_4\text{-}H_2O}$ to some of the model parameters, which are typical to three ΔG_{net} ranges:

(*i*) At small-negative ΔG_{net} ($\gtrapprox -60$ kJ mol^{-1}) the Mtr- and Mcr-catalyzed reactions depart from equilibrium, and consequently, the combination of these reactions' equilibrium and kinetic fractionation factors (EFFs and KFFs, respectively) determines the magnitude of $^{13}\varepsilon_{CO_2\text{-}CH_4}$ over this ΔG_{net} range. Thus, changes to parameters that affect the departure from equilibrium of these reactions manifest as a change in the maximal $^{13}\varepsilon_{CO_2\text{-}CH_4}$ value reached at ΔG_{net} of ≈ -40 kJ mol^{-1} (a vertical movement of the $^{13}\varepsilon_{CO_2\text{-}CH_4}$ peak, as in panels 13–15, 26, 27, 31, and 35 of fig. S14). These parameters include the K_M and V^+ values, the exact $\Delta G_r'^0$ values of the Mtr- and Mcr-catalyzed reactions ($R_{\Delta G_r'^0}$, see table S1), and the initial concentration of HS-CoB and CoM-S-S-CoB (C_i CoB). Over the same ΔG_{net} range, changes in some parameters drive a shift of the $^{13}\varepsilon_{CO_2\text{-}CH_4}$ and $^{2}\varepsilon_{CH_4\text{-}H_2O}$ trajectories along the ΔG_{net} axis, with minimal effect on the peak $^{13}\varepsilon_{CO_2\text{-}CH_4}$ and $^{2}\varepsilon_{CH_4\text{-}H_2O}$ values (as in panels 19, 20, and 29 in fig. S14 and panels 13, 19, 26, 29, 35, and 36 in fig. S15). This is mostly evident in K_M and V^+ values of the Hdr-catalyzed reaction, which is irreversible ($J_i^-/J_i^+ \rightarrow 0$) already at small-negative ΔG_{net}. Changes in the kinetic parameters of this reaction affect the steady-state concentration of HS-CoB and CoM-S-S-CoB, and consequently shifts the departure from equilibrium of the Mtr- and Mcr-catalyzed reactions along the ΔG_{net} axis. In contrast to changes that directly affect Mtr- and Mcr-catalyzed reactions, here both reactions shift together along the ΔG_{net} axis (e.g., when forward K_M values of Hdr decrease, both Mtr- and Mcr-catalyzed reactions depart from equilibrium at smaller ΔG_{net} values) and thus the position of the $^{13}\varepsilon_{CO_2\text{-}CH_4}$ value changes accordingly on the ΔG_{net} axis, but its magnitude remains similar. Lastly, over the same ΔG_{net} range, Y_{ATP} values affect both the peak $^{13}\varepsilon_{CO_2\text{-}CH_4}$ values, and the location of the $^{13}\varepsilon_{CO_2\text{-}CH_4}$ and $^{2}\varepsilon_{CH_4\text{-}H_2O}$ trajectories of departure from equilibrium along the ΔG_{net} axis (panel 36 in figs. S14 and S15). The Y_{ATP} parameter, which sets the minimal energetic requirement for ATP production, affects the Gibbs free energy of the Mtr-catalyzed reaction

($\Delta G'_{i,\mathrm{Mtr}}$, see Methods), which in turn affects the relative order of departure from equilibrium of the Mtr- and Mcr-catalyzed reactions, and the relative expressions of their respective KFFs. As the Y_{ATP} also sets the minimal threshold on methanogenic activity, increasing or decreasing its value shifts the departure from equilibrium along the ΔG_{net} axis.

(*ii*) At ΔG_{net} between ≈ -50 and ≈ -100 kJ mol^{-1}, $^{13}\varepsilon_{CO_2\text{-}CH_4}$ values are dominated by departure from equilibrium of the Ftr-catalyzed reaction. Thus, changes to the kinetic parameters of this reaction result in a shift in the location of the decrease of $^{13}\varepsilon_{CO_2\text{-}CH_4}$ values from the larger-than-equilibrium peak to their minimal values at large negative ΔG_{net} (panels 3, 4, and 22 in fig. S14). The Ftr-catalyzed reaction is not directly involved in hydrogen atom exchange, and the departure from hydrogen isotope equilibrium is controlled mostly by departure from equilibrium of the Mcr- and Hdr-catalyzed reactions, which occurs at less negative ΔG_{net} values. Consequently, over the intermediate ΔG_{net} range, $^{2}\varepsilon_{CH_4\text{-}H_2O}$ is insensitive to essentially all model parameters (fig. S15).

(*iii*) At large-negative ΔG_{net} ($\lessapprox -100$ kJ mol^{-1}), several parameters affect the minimal values of $^{13}\varepsilon_{CO_2\text{-}CH_4}$ and $^{2}\varepsilon_{CH_4\text{-}H_2O}$. Over this ΔG_{net} range, $^{13}\varepsilon_{CO_2\text{-}CH_4}$ is controlled mostly by the relative expression of the EFFs and KFFs of the Fmd- and Mtr-catalyzed reactions. Therefore, $^{13}\varepsilon_{CO_2\text{-}CH_4}$ is sensitive to the kinetic parameters of these enzymes, most notably to their V^+ values of the Fmd- and Mtr-catalyzed reactions. Mtr has the smallest V^+ value and as ΔG_{net} increases and the enzyme is saturated with substrates, its rate comes to limit the overall methanogenesis rate. A sufficient increase in the V^+ of Mtr results in colimitation of net methanogenesis rates at large ΔG_{net} values with other enzymes, e.g., the Fmd-catalyzed reaction that completely departs from equilibrium (fig. S13), thus maximizing the expression of the KFF of Fmd (panel 26 in fig. S14). A similar effect emerges also in $^{2}\varepsilon_{CH_4\text{-}H_2O}$, mostly as sensitivity to the values of K_M and V^+ of the Mtd-, Hmd, and Frh-catalyzed reactions, which control the degree of hydrogen atom mixing between H_2 and H_2O, and to the V^+ value of the Mtr-catalyzed reaction, which is rate limiting as explained above.

Data for model calibration and validation

Model calibration to laboratory culture results

We curated net carbon and hydrogen isotopic fractionations and their relations to the Gibbs free energy of the net methanogenic reaction (ΔG_{net}) from culture experiments. Among other experimental parameters, this dataset consists of experiments with varying temperature, growth phases, substrates used and microbial strains. We screened the data according to the following criteria: (*i*) including data from stationary growth phases where growth was reported; (*ii*) excluding data that may have been affected by Rayleigh (isotopic) distillation; (*iii*) excluding data that were ignored in the original publications due to known measurement errors. For each sample we used the reported ΔG_{net}, where available, or calculated it based on the reported concentrations of H_2, CO_2 and CH_4 using: $\Delta G_{net} = \Delta G_r'^0 + RT \ln Q$, where $\Delta G_r'^0$ is the standard Gibbs free energy of the reaction (corrected for the temperature with the Van't Hoff equation), R is the ideal gas constant, T is the temperature and $Q = [CH_4]/\left([CO_2][H_2]^4\right)$ is the reaction quotient. Isotopic data from experiments with small negative ΔG_{net} values were mostly obtained from co-cultures, which complicated measurements of headspace H_2 concentrations. We corrected for the concentrations of H_2 in these samples as explained in the Methods. All the samples that were used are listed in table S9.

The $^2\varepsilon_{CH_4\text{-}H_2O}$-$\Delta G_{net}$ relation at small negative ΔG_{net} values shows contradicting trajectories between two similar experiments, conducted by Yoshioka et al., 2008 (39) and by Okumura et al., 2016 (20) (henceforth Yos08 and Oku16). While both reports used the same thermophilic co-culture of *S. lipocalidus* and *M. thermoautotrophicus* strain ΔH, $^2\varepsilon_{CH_4\text{-}H_2O}$ values inferred in the two studies differ by ≈100‰ at similar ΔG_{net} values (fig. S4). These differences are at least in part due to the analytical methods used. While Yos08 oxidized CH_4 to H_2O and then reduced the H_2O to H_2, which was then introduced into an isotope-ratio mass spectrometer (IRMS), Oku16 pyrolyzed CH_4 directly to H_2, which was then introduced into an IRMS. The latter approach is considered more accurate due to a lower risk of contamination during sample processing and lesser exposure to humid air. This is one reason for which we chose to use the Oku16 over the Yos08 data. A second reason is that the Oku16 data also include measurements of both carbon and hydrogen

isotopes over a larger range of ΔG_{net} values.

There are considerable differences between $\Delta^{13}CH_3D$ values in hydrogenotrophic methanogens with and without membrane-embedded electron carriers (e.g., methanophenazine), the latter of which are the focus of our model. While $^2\varepsilon_{CH_4\text{-}H_2O}$ is of a similar range for these two groups, $\Delta^{13}CH_3D$ for methanogens with methanophenazines is between -6 and 0‰, whereas for methanogens without methanophenazines the observed range is -2 to 3‰. The laboratory culture data collected to date is insufficient to determine whether this difference is statistically significant, though there may be a physiological basis for it, as methanogens with methanophenazines have distinct metabolic characteristics that may affect the dynamics of departure from equilibrium.

Cell-specific methanogenesis rates

There are currently limited data on cell-specific methanogenesis rates in natural environments. We bridge this gap by comparing compiled bulk methanogenesis rates (bMR) and estimates of cell density. bMR values were obtained from the results of either ratiotracer experiments or reaction-diffusion models, both of which carry uncertainties. In radiotracer experiments, the rate of methanogenesis is assumed to be equal to the rate of CO_2 reduction to methane, but in fact the measured rate of CO_2 reduction serves as an upper limit on methanogenesis rates. If the reaction is close to equilibrium, then the net rate of methanogenesis will be lower than the radiotracer-based estimate, possibly by orders of magnitude if the reversibility between methane and CO_2 is higher than 0.9. Moreover, some of the radiotracer experiments are conducted under conditions that may favor higher methanogenesis rates (e.g., increased partial pressure of H_2 in the headspace), resulting in overestimation of the in-situ rates. Models of in-situ methanogenesis rates provide an estimate for the net methanogenesis rates based on the concentration and isotopic gradients of methane and DIC, but carry uncertainties due to the choice of model parameters, such as the net fractionation of carbon and hydrogen isotopes associated with methanogenesis.

To estimate the abundance of cells, where no measurements exist, we used a general relation between cell density and depth within the sediment in marine environments (90, 91): $y = 7.73 \cdot$

$10^7 \times z^{-0.6332}$, where z is the depth in meters and y is the number of cells per cubic centimeter of sediment. We assume that of these cells 12% are Archaea in open-ocean sites and 40% in ocean margin sites (92), and that 50% of Archaea are methanogens (91).

Supplementary Figures

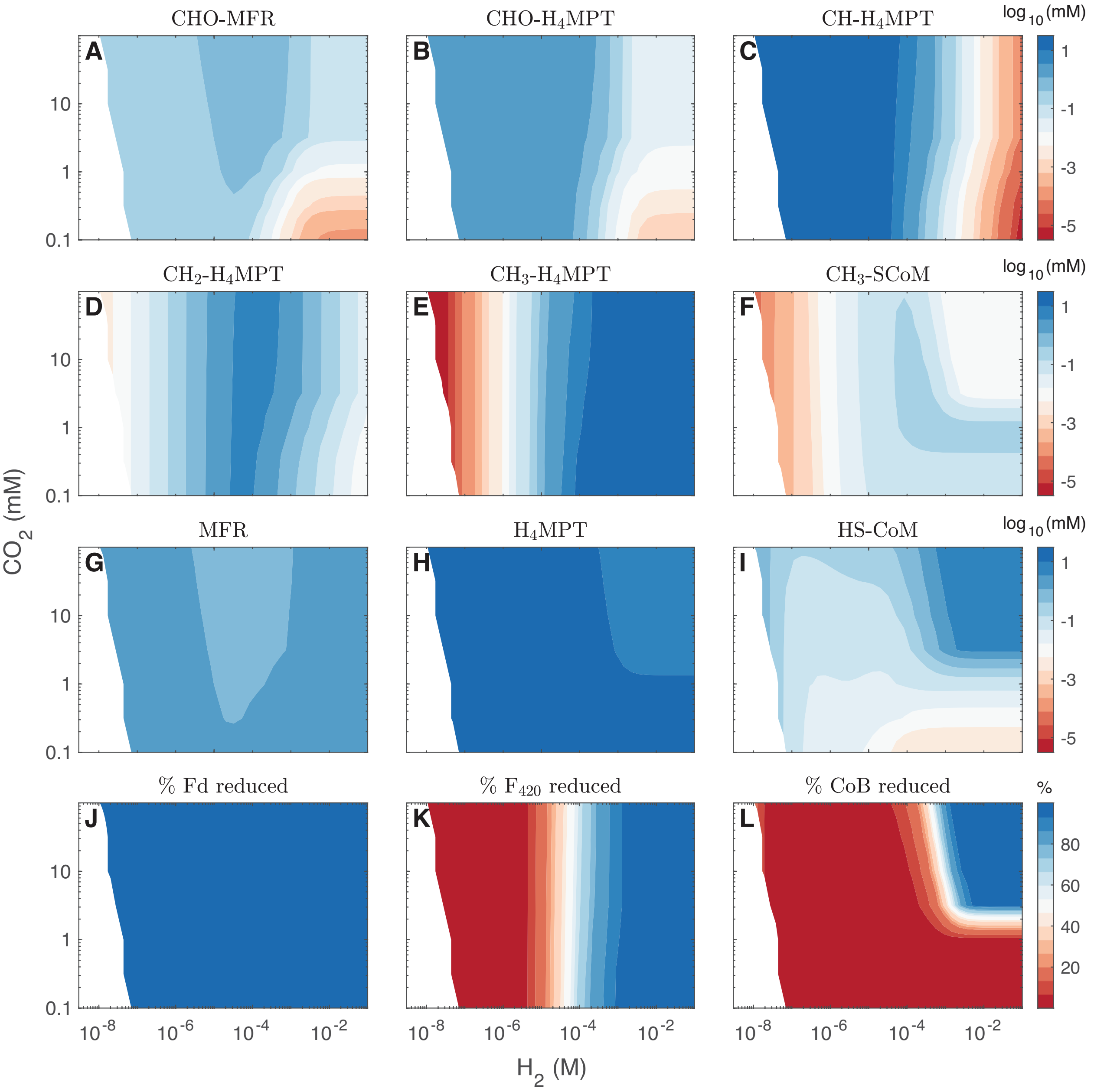

Figure S1: Intracellular metabolite concentrations. (**A-I**) $\log_{10}$ of concentrations in mM (see panel color legends for each row in the right-most panel). (**J-L**) Percentage of electron carriers in reduced form.

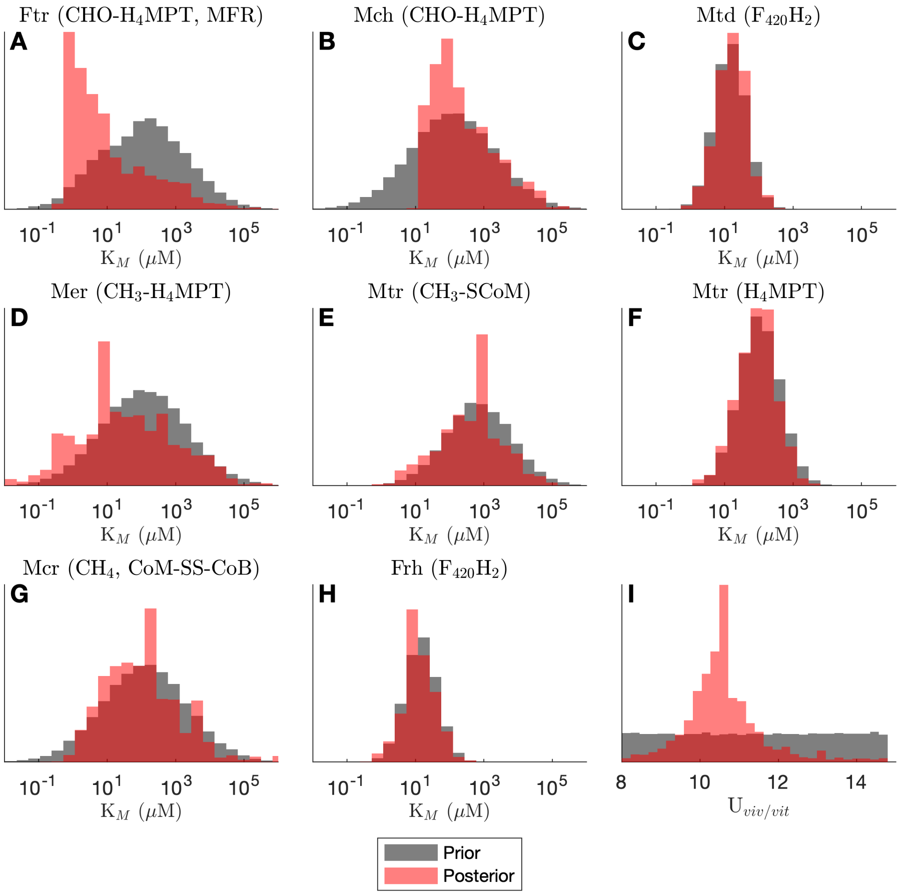

Figure S2: Prior and posterior distributions of metabolic model parameters. (**A–H**) K_m values and (**I**) $U_{(viv/vit)}$, for 10^6 simulations. An overview of the prior and posterior distributions is in table S5.

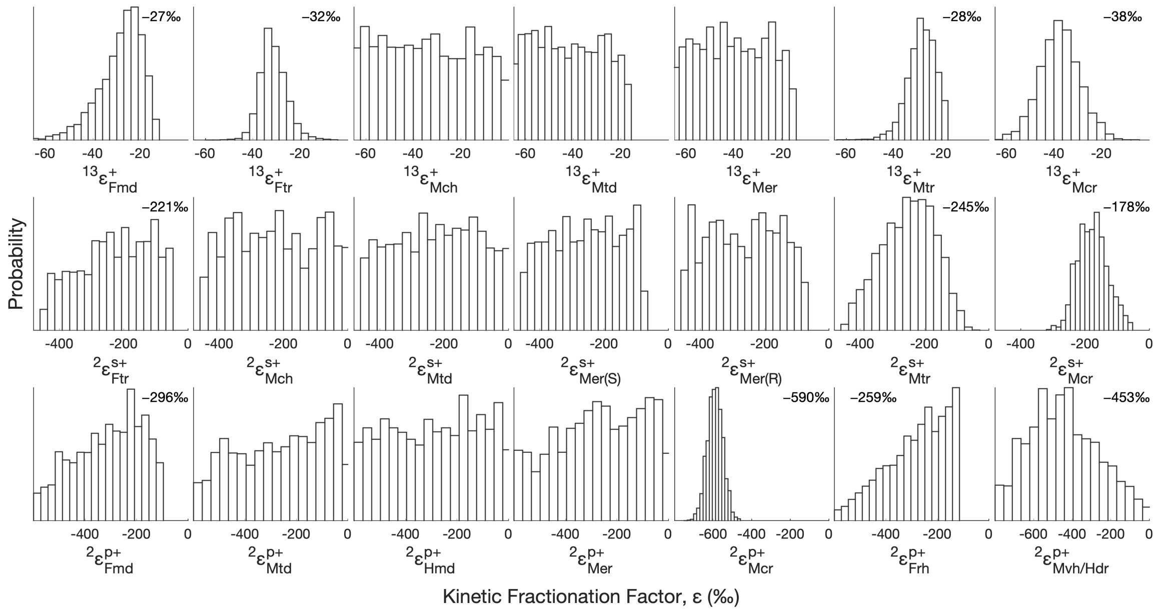

Figure S3: Posterior distributions of kinetic isotope fractionation factors (KFFs). The posterior KFF distributions were generated by weighting 10^6 individual KFF value combinations drawn from uniform prior distributions by the inverse of the square of the sum of squared model-measurement mismatches ($1/SSE^2$). Here we assumed that KFFs are similar in both mesophilic and thermophilic conditions. Values are plotted in permil (‰), where $\varepsilon = \alpha - 1$. Carbon isotope KFFs (top row) were drawn from uniform prior distributions with $\alpha^+ \in (0.935, 1)$. The KFF of Mcr was assigned a normal prior distribution with a mean of 0.9615 and a standard deviation of 0.01 (42). Secondary hydrogen isotope KFFs (middle row) were drawn from uniform prior distributions with $\alpha^+ \in (0.6, 1)$, and primary hydrogen isotope KFFs (bottom row) were drawn from prior uniform distributions with $\alpha^+ \in (0.4, 1)$ ($\alpha^+ \in (0.2, 1)$ for the Mvh/Hdr-catalyzed reaction). The primary hydrogen isotope KFFs of Mcr were drawn from normal prior distributions with a mean of 0.41 and a standard deviation of 0.04 (42). The secondary hydrogen isotope KFFs of Mcr were drawn from normal prior distributions with a mean of 0.85 and a standard deviation of 0.035. The values in the upper right corner of the panels represent the median posterior KFF values. In simulations where the posterior distribution is similar to the prior (uniform) distribution we do not show these median values. Primary and secondary hydrogen isotope KFFs are denoted by superscripted 'p' and 's' respectively.

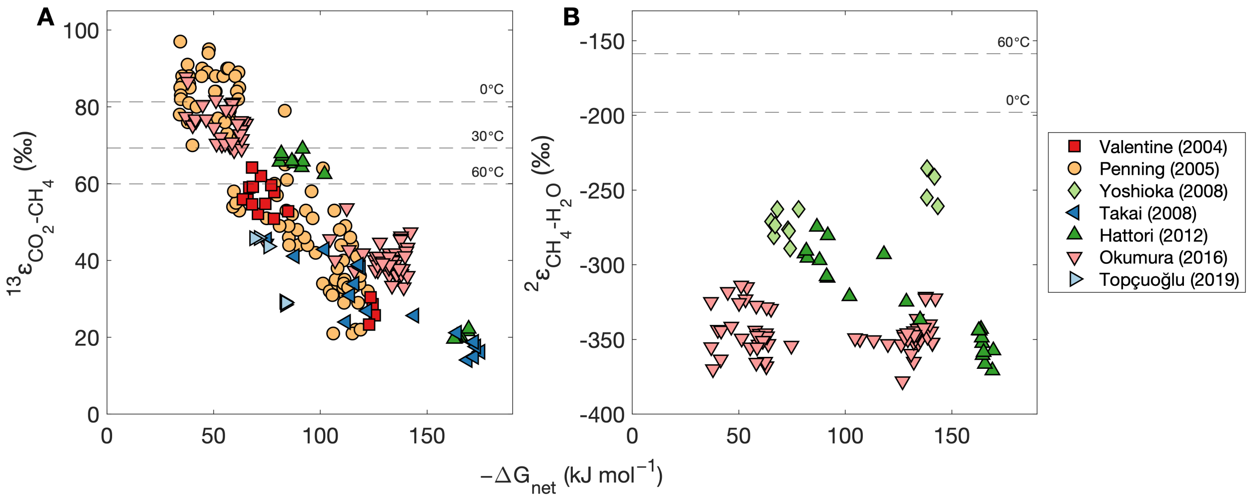

Figure S4: Isotopic fractionation in laboratory cultures depends on the thermodynamic drive. A compilation of (**A**) carbon isotope fractionation between CO_2 and CH_4 ($^{13}\varepsilon_{CO_2\text{-}CH_4}$), and (**B**) the hydrogen isotope fractionation between CH_4 and H_2O ($^{2}\varepsilon_{CH_4\text{-}H_2O}$). The dashed lines represent the EFFs for the temperatures that are noted next to the lines, calculated at the M06-L/def2-TZVP level of theory with the SMD implicit solvation model (43). The data (16–21, 39) are listed in table S9.

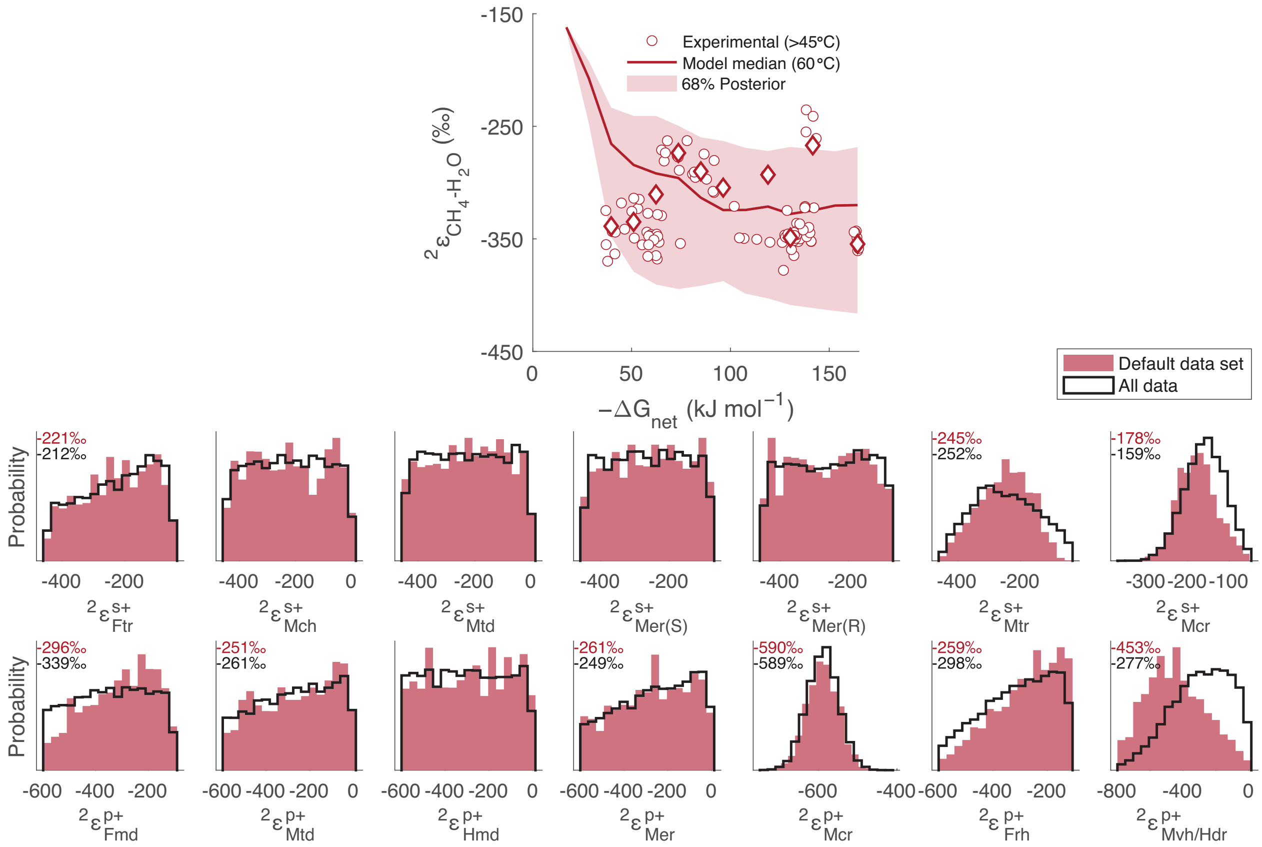

Figure S5: Calibration of hydrogen isotope kinetic fractionation factors. Top: model results for calibration against data (circles) from Yoshioka et al., 2008 (39), Hattori et al., 2012 (19), and Okumura et al., 2016 (20). The diamonds are the binned averages that the model was calibrated against. Middle and lower rows are the posterior KFF distributions for the data from Okumura et al., (red histograms) and for the three data sets combined (black line). The respective median values of the KFFs (where the posterior distribution is not uniform) are noted in the upper left corner of each panel.

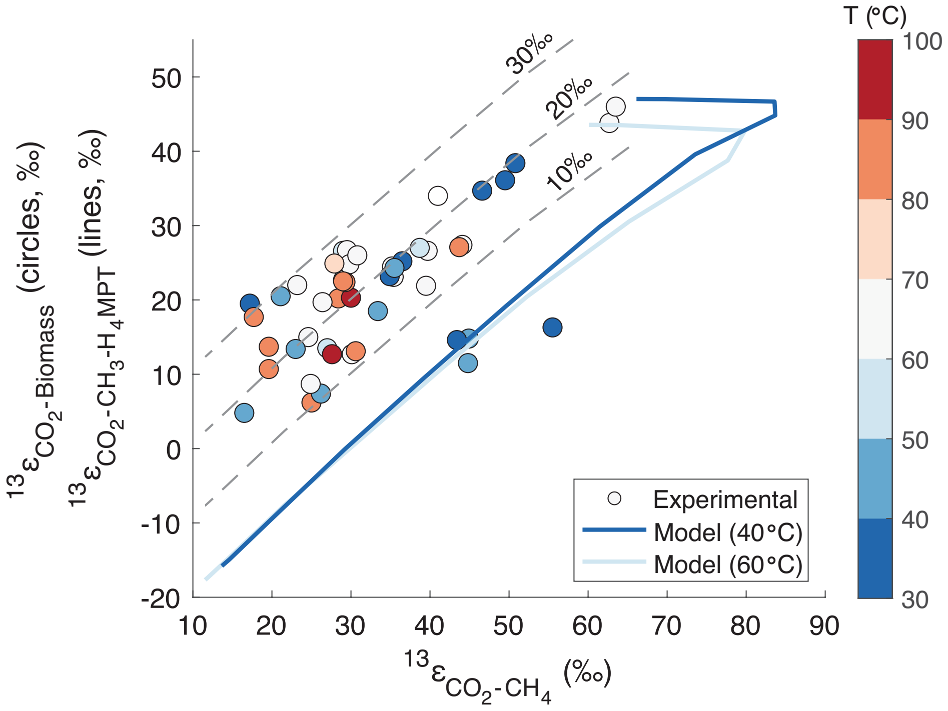

Figure S6: Carbon isotope fractionation between CO_2, CH_3-H_4MPT, and biomass. Lines show the median model results for the CO_2–CH_3-H_4MPT carbon isotope fractionation ($\varepsilon_{CO_2\text{-}CH_3}$), at 40 and 60°C, and [$CO_2$] of 1 mM. The circles are experimentally measured values of the CO_2–biomass carbon isotope fractionation, as previously compiled (49, 51). The colors of the circles correspond to the experimental temperature, and the dashed lines represent 10, 20 and 30‰ offsets from $\varepsilon_{CO_2\text{-}CH_3}$ on the y-axis.

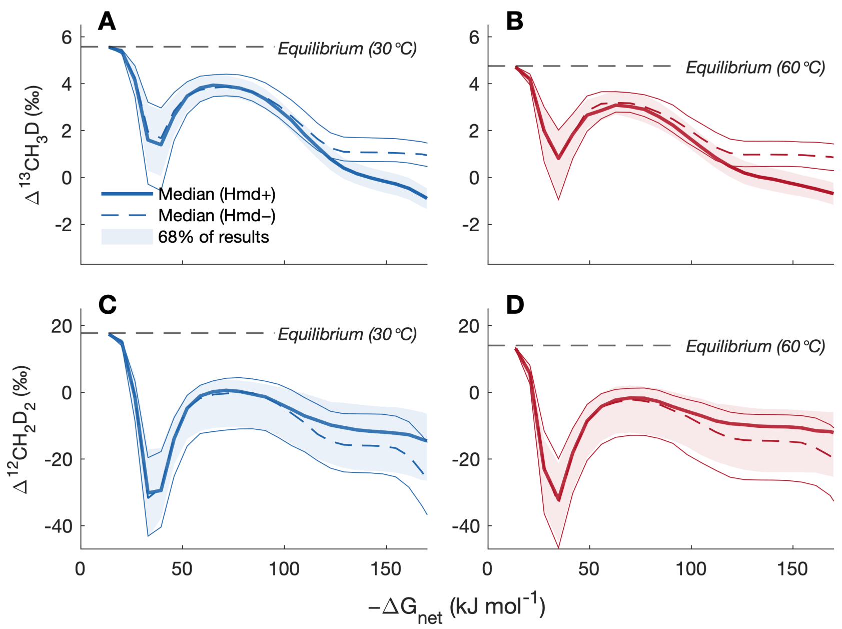

Figure S7: Clumped isotopologue abundances. Model clumped isotope results of 200 simulations at 30°C (blue) and 60°C (red) against ΔG_{net}. The thick red and blue lines show the median, and the envelopes show 68% of the model results with Hmd activity. The dashed red and blue lines and the envelopes denoted by thin lines show the median and 68% of the model results without Hmd activity. The gray lines represent temperature-dependent isotopic equilibrium. (**A-B**) $\Delta^{13}CH_3D$. (**C-D**) $\Delta^{12}CH_2D_2$.

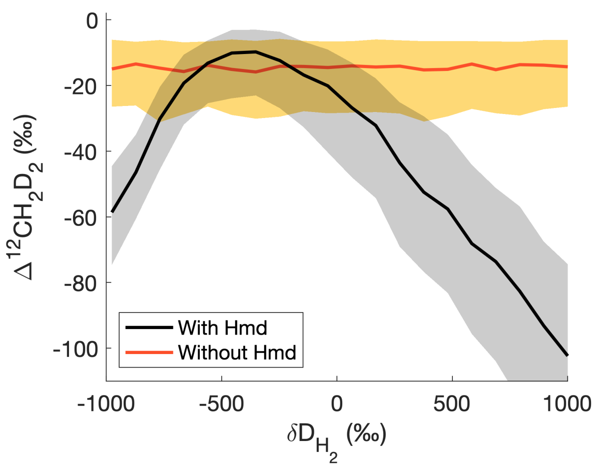

Figure S8: A combinatorial effect in hydrogenotrophic methanogenesis due to activity of Hmd. Methane $\Delta^{12}CH_2D_2$ values against H_2 δD values calculated at 60°C, at $[H_2]$ of 50 mM. The δD values of H_2O in all the calculations are 0‰. The black and orange lines are medians of 10^4 simulations with and without the Hmd-catalyzed reaction, respectively, and the envelopes contains 68% of the results. The variability in the $\Delta^{12}CH_2D_2$ values is due to the hydrogen isotope KFFs, which were randomly drawn from the posterior distributions presented in fig. S3, with a constant KFF of 0.95 for Hmd.

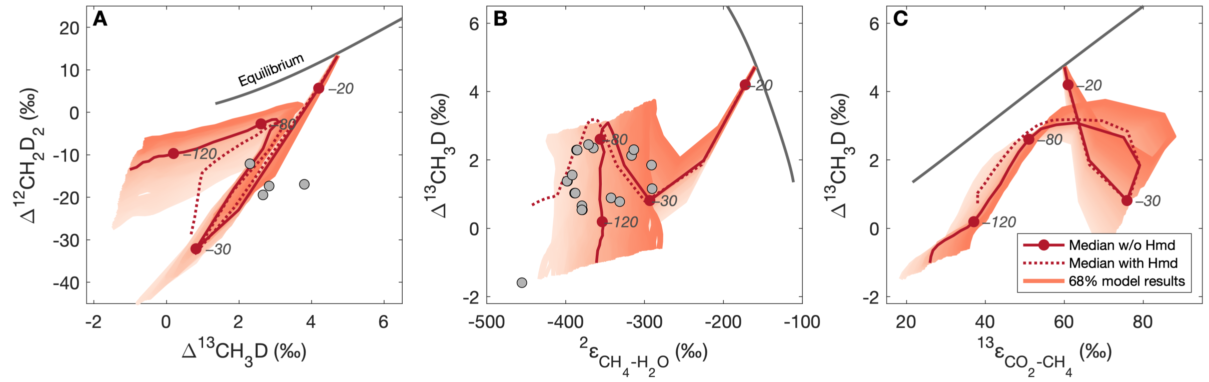

Figure S9: Clumped isotopologue abundances for thermophilic conditions. Experimental data (gray circles) and model results (lines and envelopes) of 200 simulations at 60°C. The dark gray lines represent temperature-dependent isotopic equilibrium at 0-350°C, and the thick red lines show the median of the simulations, with red circles at ΔG_{net} values of -20, -30, -80, and -120 kJ mol^{-1}. (**A**) $\Delta^{13}CH_3D$ against $\Delta^{12}CH_2D_2$. (**B**) $\Delta^{13}CH_3D$ against $^2\varepsilon_{CH_4\text{-}H_2O}$. (**C**) $\Delta^{13}CH_3D$ against $^{13}\varepsilon_{CO_2\text{-}CH_4}$. Laboratory culture samples are from hydrogenotrophic methanogens that do not have membrane-associated methanophenazines.

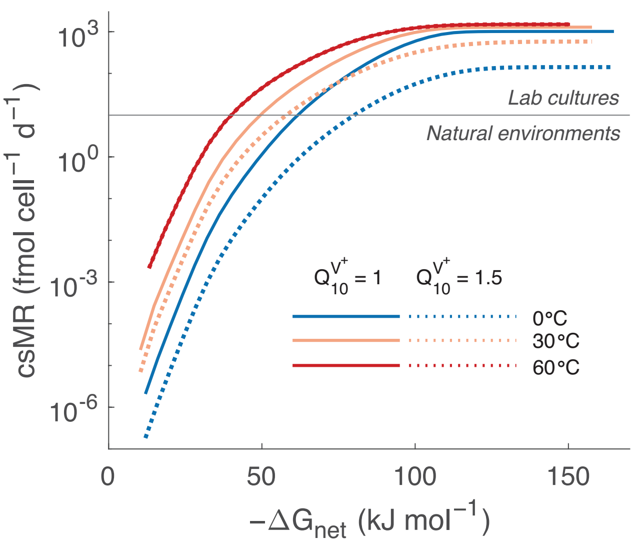

Figure S10: The dependence of cell-specific methanogenesis rate (csMR) on ΔG_{net}**.** The solid lines show the default laboratory-calibrated model results for the same temperatures with Q_{10}^{V+} of 1, and the dotted lines show the results for Q_{10}^{V+} of 1.5. The calculations are for [CO_2] and [CH_4] of 1 mM. The cell volume is 1 μm^3.

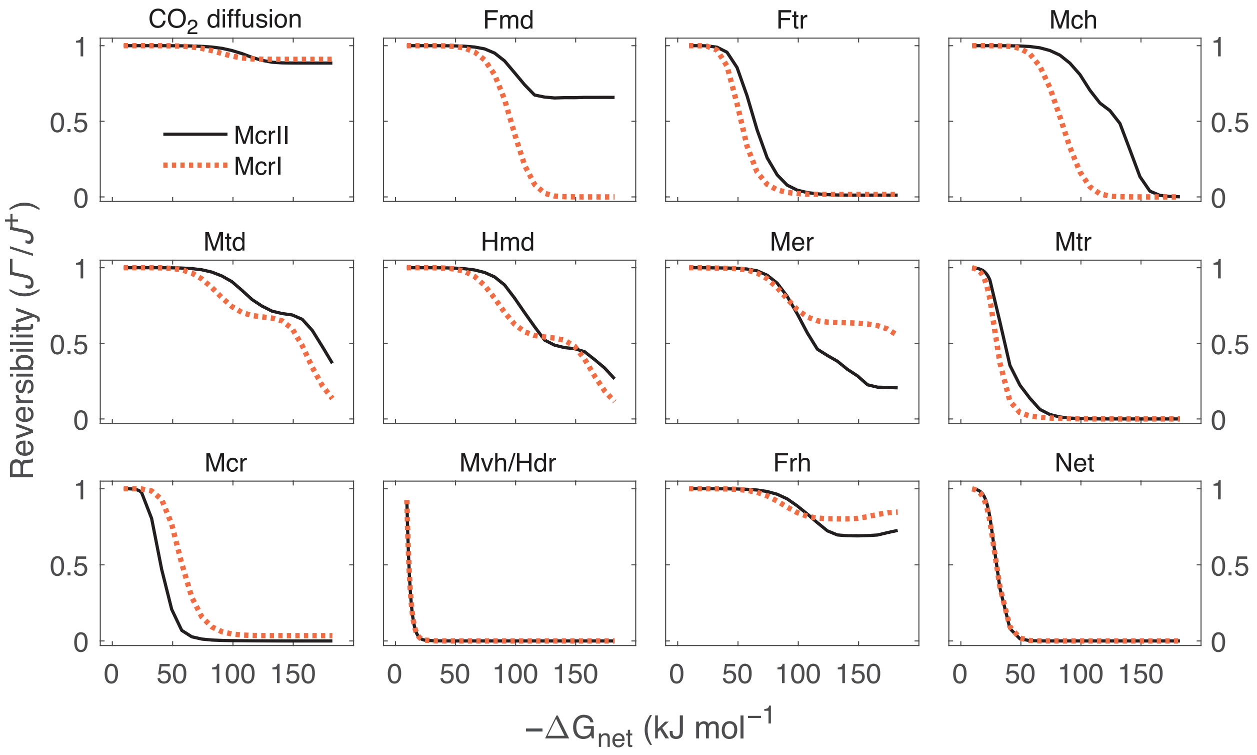

Figure S11: Reversibility against the thermodynamic drive (ΔG_{net}) **of methanogenesis under energy-limiting conditions.** Mcr II results simulate growth under optimal conditions in lab cultures (black), and Mcr I is for growth in energy-limited conditions (orange). The solid lines are the median of 100 model simulations The reversibility, defined as the ratio of the backward to forward reaction rates of an individual reaction (J_i^-/J_i^+) ranges from 1 (full reversibility) to 0 (unidirectional reaction).

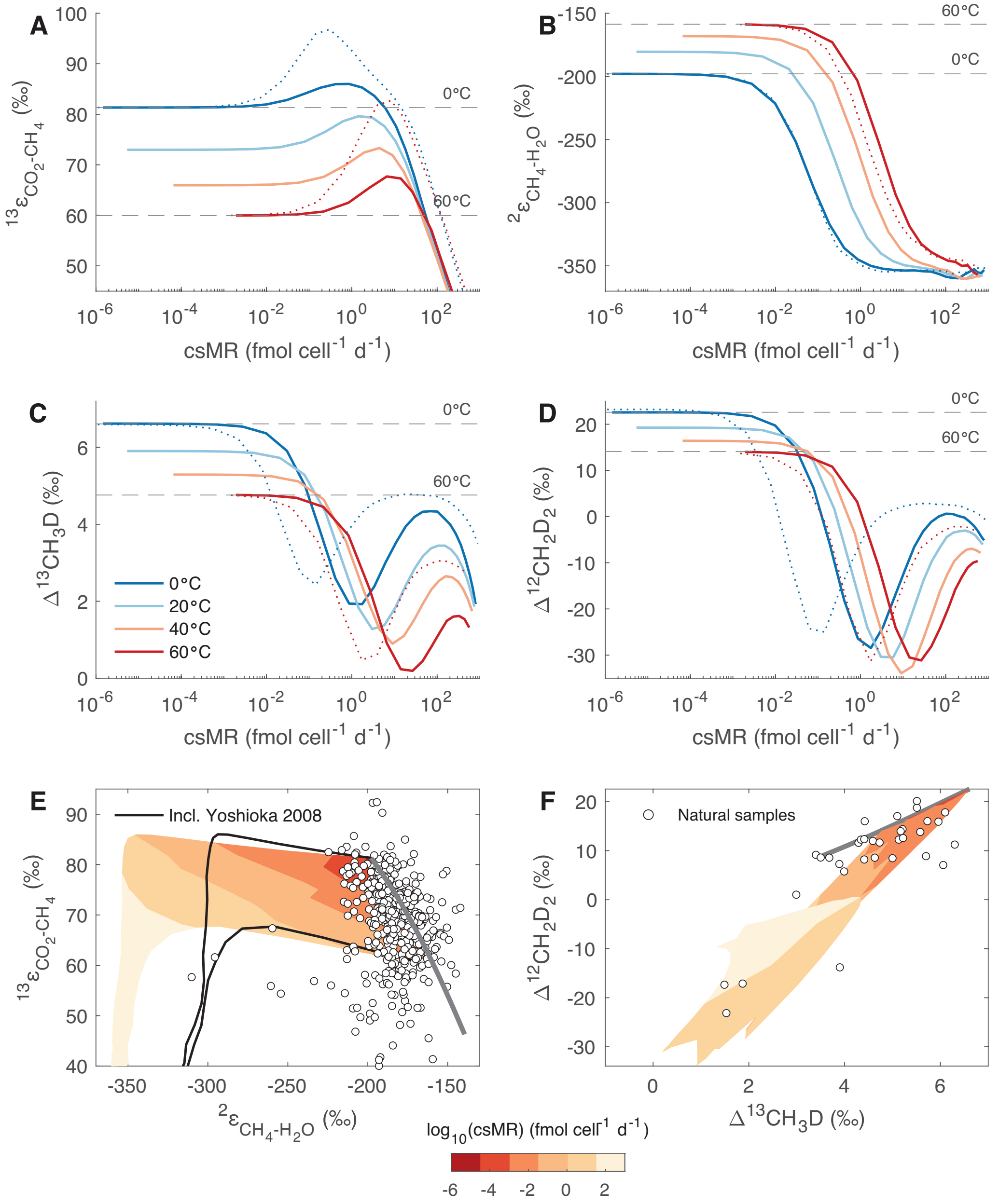

Figure S12: Isotopic fractionation during methanogenesis in energy-limited conditions. The dependence on csMR of (**A**) $^{13}\varepsilon_{CO_2\text{-}CH_4}$, (**B**) $^{2}\varepsilon_{CH_4\text{-}H_2O}$, (**C**) $\Delta^{13}CH_3D$, and (**D**) $\Delta^{12}CH_2D_2$. The dashed gray lines in panels **A-D** represent the equilibrium isotopic fractionations between 0°C and 60°C. (**E**) Co-variation of $^{13}\varepsilon_{CO_2\text{-}CH_4}$ and $^{2}\varepsilon_{CH_4\text{-}H_2O}$. The black lines indicate the co-variation of $^{13}\varepsilon_{CO_2\text{-}CH_4}$ and $^{2}\varepsilon_{CH_4\text{-}H_2O}$ with KFFs from posterior distributions that were calibrated against a dataset including results from Yoshioka et al., 2008 (39). (**F**) Co-variation of $\Delta^{12}CH_2D_2$ and $\Delta^{13}CH_3D$. The contours in panels **E-F** are the $\log_{10}$ of the csMR as predicted by our metabolic model between 0°C and 60°C, and the circles are biogenic environmental samples (table S10). In panels **A-D**, the dotted lines show the default laboratory-calibrated model results for the same temperatures (Mcr II). The calculations are for $[H_2]$ in the range of 1 nM to 5 μM, and $[CO_2]$ and $[CH_4]$ of 1 mM. The cell volume is 1 μm^3.

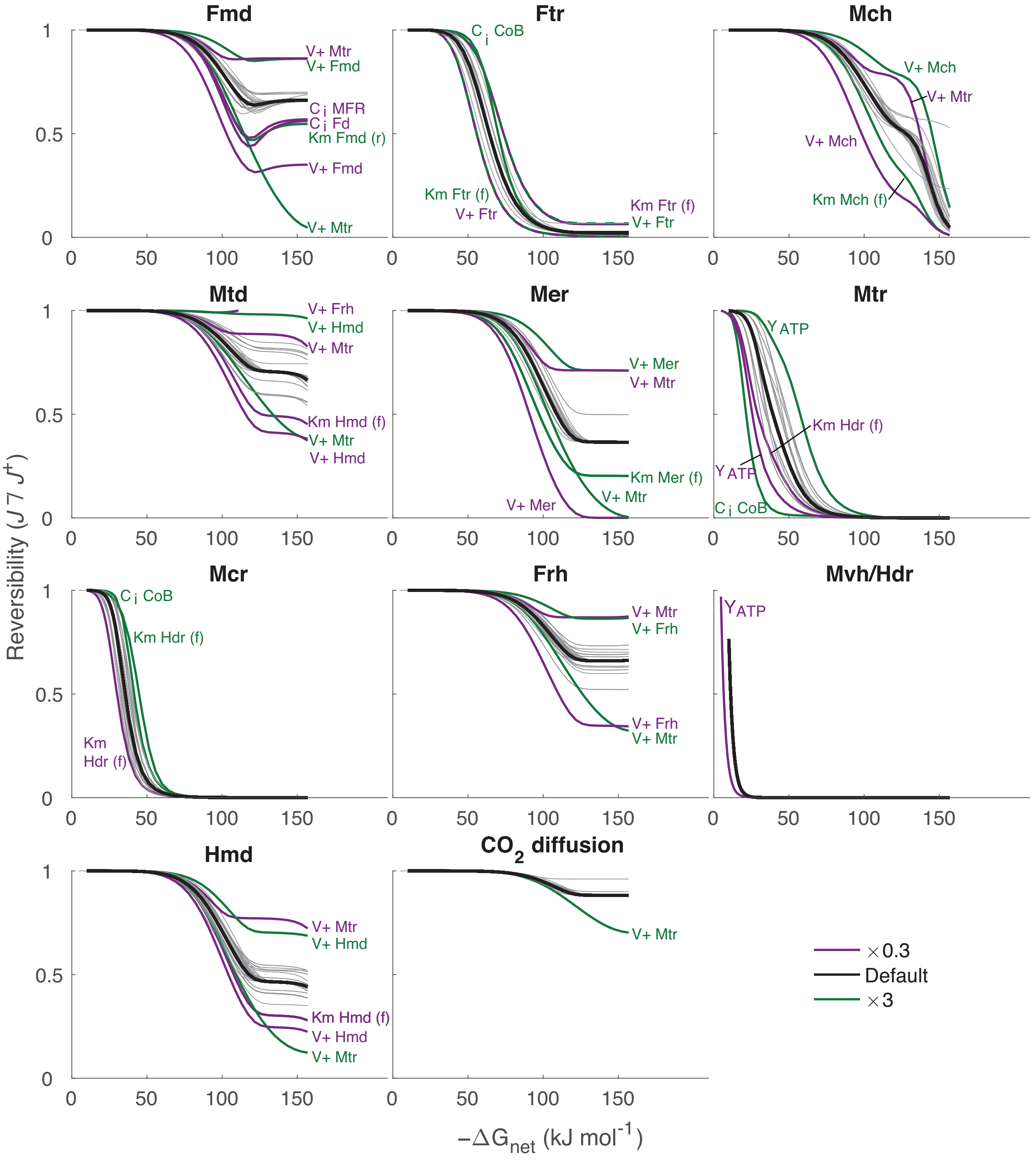

Figure S13: Sensitivity of reaction reversibility to model parameters. Black lines represent results with the default parameters, purple and green lines represent a decrease or an increase by a factor of three to a default parameter value, respectively. The parameter names are written next to their respective reversibility trajectories. For K_M values, the 'f' and 'r' notations denote forward and reverse direction of the reaction, respectively. The 'C_i' denote initial concentrations of a metabolite. The gray trajectories are for parameters that had little or no effect on the reversibility.

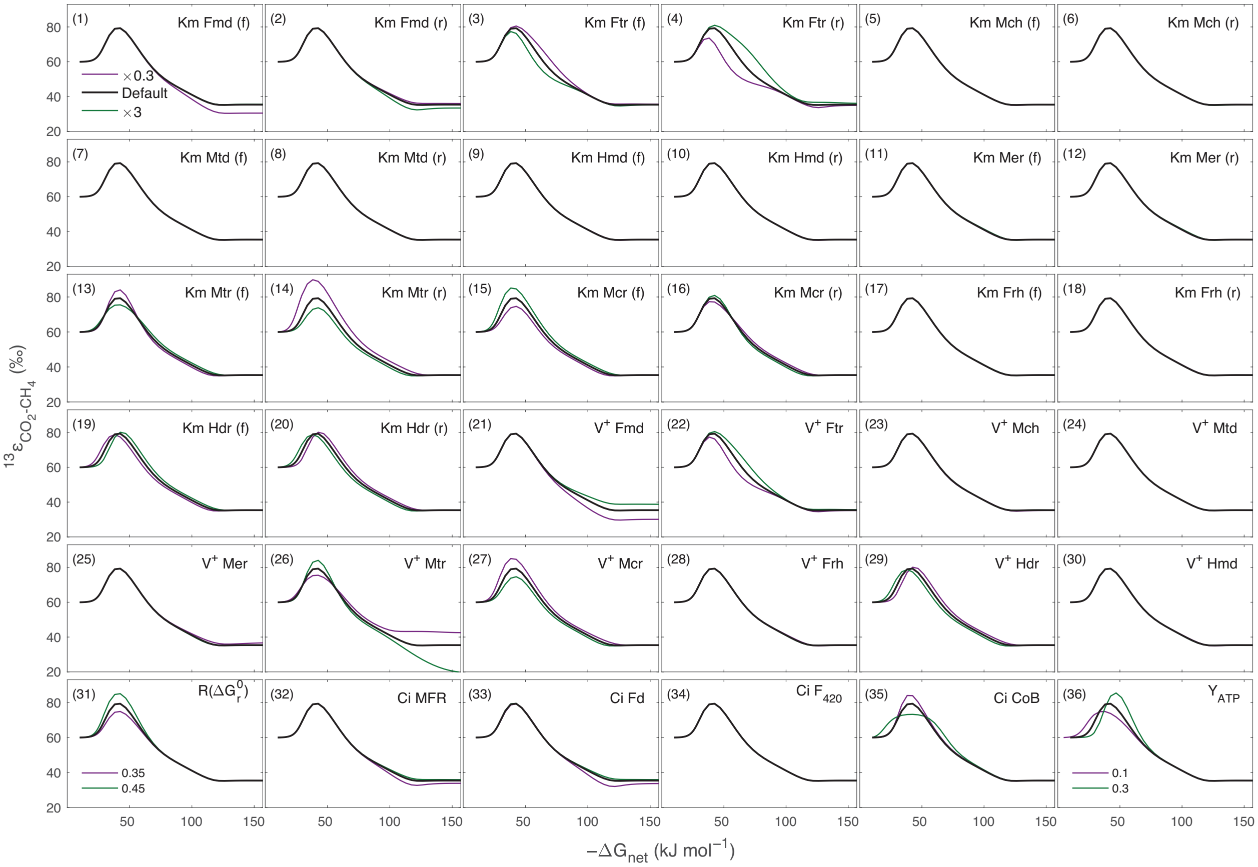

Figure S14: Sensitivity of CO_2–CH_4 carbon isotope fractionation to model parameters. Black lines represent results with the default parameters, purple and green lines represent a decrease or an increase by a factor of three to the default parameter, respectively, unless otherwise noted in the panel. For K_M values, the 'f' and 'r' denote forward and reverse direction of the reaction, respectively. The 'C_i' denote initial concentrations of a metabolite. The KFFs used are the median values from the posterior distributions in fig. S3.

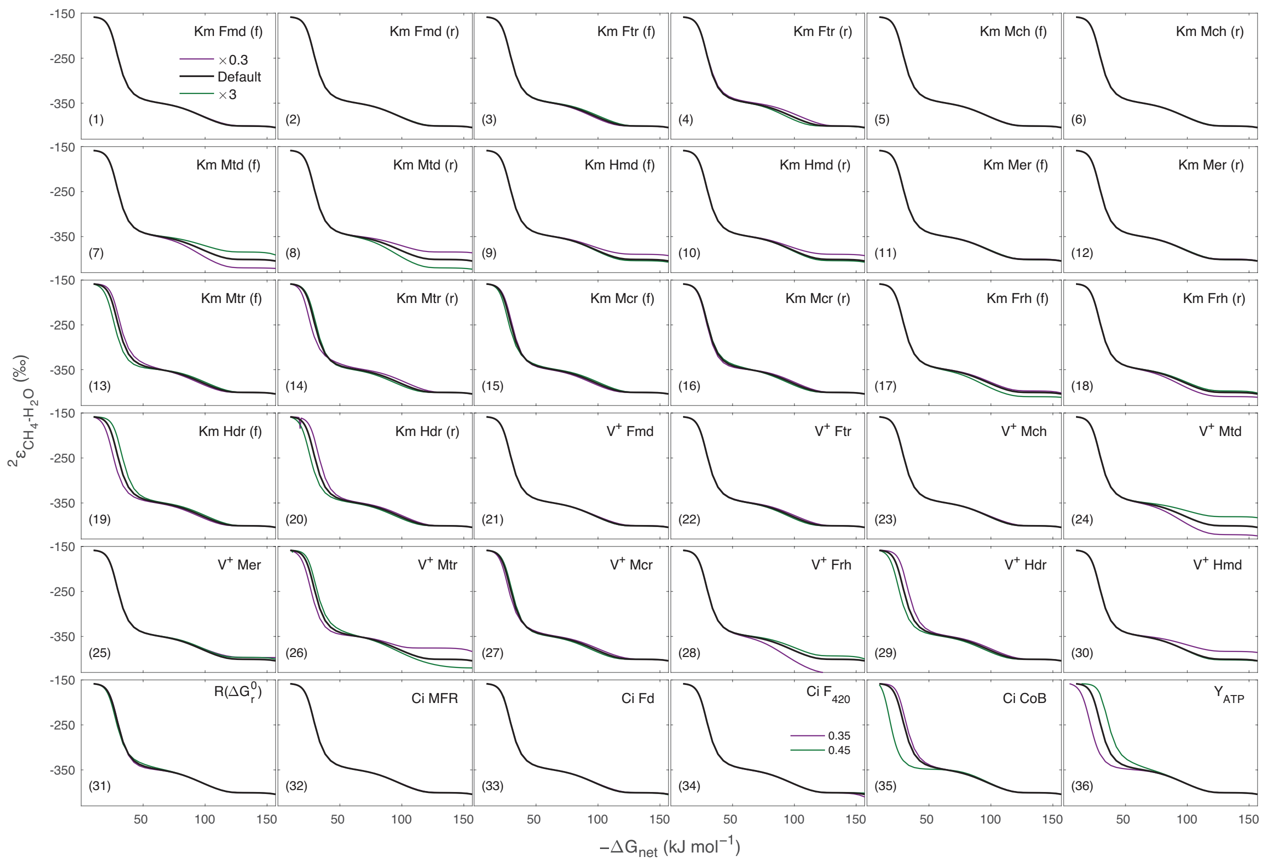

Figure S15: Sensitivity of hydrogen isotope fractionation to model parameters. Black lines represent results with the default parameters, purple and green lines represent a decrease or an increase by a factor of three to the default parameter, respectively, unless otherwise noted in the panel. For K_M values, the 'f' and 'r' denote forward and reverse direction of the reaction, respectively. The 'C$_i$' denote initial concentrations of a metabolite. The KFFs used are the median values from the posterior distributions in fig. S3.

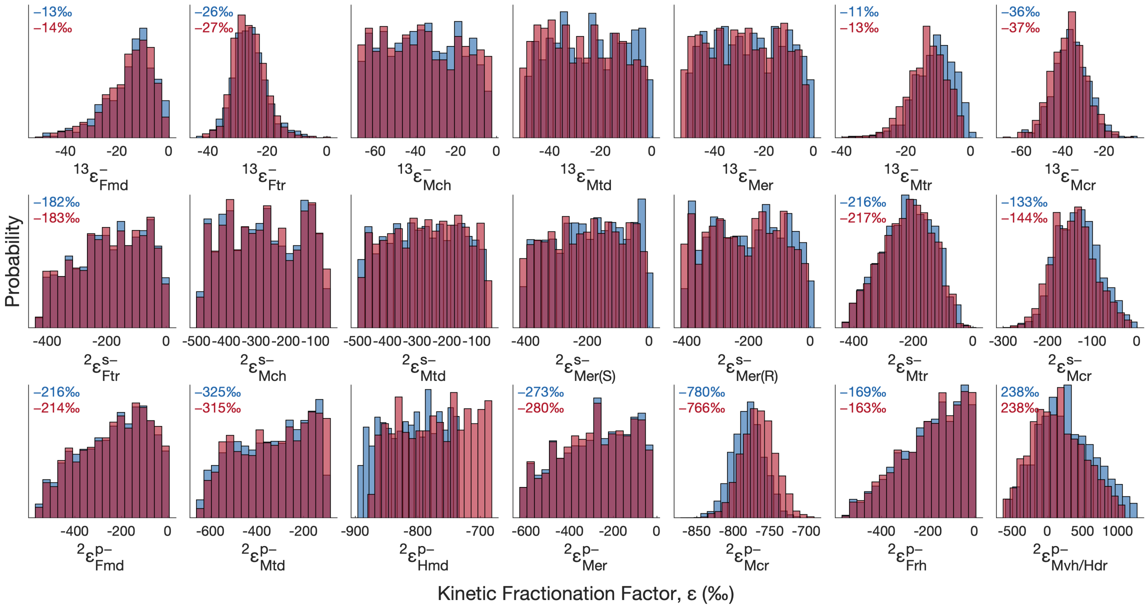

Figure S16: Posterior distributions of reverse kinetic isotope fractionation factors (KFFs). The posterior reverse KFF distributions are derived from the ratio $\alpha^{-} = \alpha^{\text{eq}} \times \alpha^{+}$, where α^{eq} is the temperature-dependent EFF of each reaction, and α^{+} is the forward KFF from distributions in fig. S3, for mesophilic (blue) and thermophilic (red) conditions. The values in the upper-left of the panels represent the median posterior KFF values for mesophilic (blue) and thermophilic (red) conditions. In simulations where the posterior distribution is similar to the prior (uniform) distribution we do not show these median values. Primary and secondary hydrogen isotope KFFs are denoted by superscripted 'p' and 's' respectively.

Supplementary Tables

Table S1: The Gibbs free energy of the reactions in hydrogenotrophic methanogenesis. We used the transformed Gibbs free energy ($\Delta G_r'^0$), which is the Gibbs free energy at a particular pH and ionic strength (here at pH 7) (27). For redox reactions, we calculated $\Delta G_r'^0$ of the half-reactions using the Nernst equation with $\Delta G_r'^0 = -nFE'^0$, where n is the numbers of electrons transferred, F is the Faraday constant and E'^0 is the standard redox potential (table S4). We note that the temperature dependence of $\Delta G_r'^0$ values is not available, other than that of the net methanogenic reaction.

	Enzyme	Reaction			$\Delta G_r'^0$ (kJ mol^{-1})	Ref./Source
		$4H_{2(aq)} + CO_{2(aq)}$	$\rightleftharpoons$	$CH_{4(aq)} + 2H_2O$	−188.8	†
		$CO_{2(out)}$	$\rightleftharpoons$	$CO_{2(in)}$		
1	Fmd	$CO_{2(aq)}$ + MFR + Fd^{2-}_{red}	$\rightleftharpoons$	CHO-MFR + Fd_{ox}	10.3	(93, 94)
2	Ftr	CHO-MFR + H_4MPT	$\rightleftharpoons$	CHO-H_4MPT + MFR	−3.5	(95)
3	Mch	CHO-H_4MPT	$\rightleftharpoons$	CH≡H_4MPT$^+$ + H_2O	−4.2	(95)
4a	Mtd	CH≡H_4MPT$^+$ + $F_{420}H_2$	$\rightleftharpoons$	CH_2=H_4MPT + F_{420}	2.0	(95)
4b	Hmd	CH≡H_4MPT$^+$ + $H_{2(aq)}$	$\rightleftharpoons$	CH_2=H_4MPT	−26	§
5	Mer	CH_2=H_4MPT + $F_{420}H_2$	$\rightleftharpoons$	CH_3-H_4MPT + F_{420}	−1.7	(95)
6	Mtr	CH_3-H_4MPT + HS-CoM	$\rightleftharpoons$	CH_3-SCoM + H_4MPT	−26.9	¶
7	Mcr	CH_3-SCoM + HS-CoB	$\rightleftharpoons$	$CH_{4(aq)}$ + CoM-S-S-CoB	−17.9	¶
8	Frh	F_{420} + $H_{2(aq)}$	$\rightleftharpoons$	$F_{420}H_2$	−28.0	(32)
9	Mvh/Hdr	$2H_{2(aq)}$ + CoM-S-S-CoB + Fd_{ox}	$\rightleftharpoons$	HS-CoM + HS-CoB + Fd^{2-}_{red}	−90.8	(93, 96)

Abbreviations: F_{420}, coenzyme F_{420}; Fd, ferredoxin; H_4MPT, tetrahydromethanopterin; HS-CoB, coenzyme B; HS-CoM, coenzyme M; MFR, methanofuran; Fmd, formyl-MFR dehydrogenase; Ftr, formyltransferase; Mch, methenyl cyclohydrolase; Mtd, methylene-H_4MPT dehydrogenase; Hmd, H_2-dependent methylene-H_4MPT dehydrogenase; Mer, methylene-H_4MPT reductase; Mtr, methyl transferase, Mcr, methyl-CoM reductase; Frh, F_{420} reducing hydrogenase; Mvh/Hdr, methylviologen hydrogenase/heterodisulfide reductase.

† Net methanogenesis at 60°C, calculated by Van't Hoff's Equation. Whenever possible, $\Delta G_r'^0$ for individual reactions were at 60°C.

§ Internally consistent value, inferred from summation of reactions 4 and 8 in this table.

¶ Reactions 6 and 7 each have an estimated $\Delta G_r'^0$ of -30 ± 10 kJ mol^{-1} (with CH_4 in the gaseous phase). We allocate the exact $\Delta G_r'^0$ of reactions 6 and 7 by splitting the remaining energy (i.e., the $\Delta G_r'^0$ of the total pathway minus the sum of $\Delta G_r'^0$ of reactions 1–5 and 8–9, denoted by $\Delta G_{res}'^0$), by a factor $R_{\Delta G_r'^0}$ with a default value of 0.4, such that $\Delta G_{Mcr}'^0 = \Delta G_{res}'^0 \times R_{\Delta G_r'^0}$ and $\Delta G_{Mtr}'^0 = \Delta G_{res}'^0 \times (1 - R_{\Delta G_r'^0})$.

Table S2: Metabolic model kinetic parameters.

V^+ (mM s^{-1})	**Metabolite**	K_M (mM)	**Ref/Source**	**Notes**
**1. formyl-MFR dehydrogenase (Fmd): CO_2 + MFR + Fd_{red} ⇄ CHO-MFR + Fd_{ox}**				
6.09			(97)	Inferred from $V^+/V^- = 25$
	CO_2, MFR, Fd_{red}	6.800	(93)	
	CHO-MFR	0.030	(97, 98)	Harmonic mean of two values
	Fd_{ox}	0.010		Estimate based on methylviologen as electron carrier
**2. formyl transferase (Ftr): CHO-MFR + H_4MPT ⇄ CHO-H_4MPT + MFR**				
28.53			(99)	
	CHO-MFR	0.050	(100)	Harmonic mean of two values
	H_4MPT	0.060	(100)	Estimate based on *M. barkeri*
	CHO-H_4MPT, MFR	0.005	‡	
**3. methenyl cyclohydrolase (Mch): CHO-H_4MPT ⇄ CH-H_4MPT**				
4.03			(101)	
	CHO-H_4MPT	0.148	‡	
	CH-H_4MPT	0.167	(102, 103)	Estimate based harmonic mean of two values from *M. barkeri* and *M. kandleri*
**4a. methylene dehydrogenase (Mtd): CH-H_4MPT + $F_{420}H_2$ ⇄ CH_2-H_4MPT + F_{420}**				
14.69			(104, 105)	Harmonic mean of two values
	CH-H_4MPT	0.050	(106)	
	$F_{420}H_2$	0.016	‡	
	CH_2-H_4MPT	0.033	(105)	
	F_{420}	0.065	(105)	
**4b. H_2-producing methylene dehydrogenase (Hmd): CH-H_4MPT + H_2 ⇄ CH_2-H_4MPT**				
45.65			(107)	
	CH-H_4MPT	0.050	(106)	
	H_2	0.150	(108)	
	CH_2-H_4MPT	0.040	(106)	
**5: methylene reductase (Mer): CH_2-H_4MPT + $F_{420}H_2$ ⇄ CH_3-H_4MPT + $F_4$20**				
6.09			(109)	
	CH_2-H_4MPT	0.300	(109)	Estimate based on *M. marburgensis*
	$F_{420}H_2$	0.003	(109)	Estimate based on *M. marburgensis*
	CH_3-H_4MPT	0.024	‡	
	F_{420}	0.040	(110)	Estimate based on *M. barkeri*

Table continued on next page.

V^+ (mM s^{-1})	Metabolite	K_M (mM)	Ref/Source	Notes
**6. methyl transferase (Mtr): CH_3-H_4MPT + HS-CoM ⇄ CH_3-S-CoM + H_4MPT**				
2.38			(111)	Original value measured at 37°C and was multiplied ×2.5
	CH_3-H_4MPT	0.135	(112)	Estimate based on *M. acetivorans*
	HS-CoM	0.277	(112)	Estimate based on *M. acetivorans*
	CH_3-S-CoM	0.559	‡	
	H_4MPT	0.098	‡	
**7. methyl-CoM reductase (Mcr): CH_3-S-CoM + HS-CoB ⇄ CH_4 + CoM-S-S-CoB**				
10.41 (76.08†)			(66, 88, 113)	Harmonic mean of three values
	CH_3-S-CoM	0.821 (0.280†)	(88, 114)	Harmonic mean of three values
	HS-CoB	0.204 (0.075†)	(88, 115, 116)	Harmonic mean of three values
	CH_4, CoM-S-S-CoB	0.110		
**8. F_{420} hydrogenase (Frh): F_{420} + H_2 ⇄ $F_{420}H_2$**				
14.15 (28.3†)			(66, 117)	Original value measured at 32°C and was multiplied by ×3.1
	H_2	0.012	(117)	
	F_{420}	0.036	(117)	
	$F_{420}H_2$	0.012	‡	
**9. methyl viologen hydrogenase/heterodisulfide reductase (Mvh/Hdr): CoM-S-S-CoB + Fd_{ox} + $2H_2$ ⇄ HS-CoM + HS-CoB + Fd_{red}**				
19.02 (57.06†)			(28, 66)	
	H_2	0.030	(28, 118)	
	CoM-S-S-Co B	0.145	(118)	
	Fd_{ox}	0.010	(119)	Estimate based on *M. barkeri*
	HS-CoM	0.200	(120)	
	HS-CoB	0.200	(120)	
	Fd_{red}	0.075	(119)	

‡ Median values drawn from the posterior distributions (see also table S5).

† Values that were for simulating methanogenesis in H_2-limited conditions.

Table S3: Metabolic model results compared to available measurements of metabolite concentrations and the oxidation state of electron carriers (represented as percent reduced). The oxidation state of CoB is calculated by $\frac{[\text{HS-CoB}]}{[\text{CoM-SS-CoB}][\text{HS-CoB}]}$, and the oxidation state of F_{420} by $\frac{[F_{420}H_2]}{[F_{420}H_2][F_{420}]}$. The data were obtained at $[H_2] \approx 10\ \mu$M, and the experiments were conducted on *M. thermoautotrophicus* at 60 °C in batch cultures, unless otherwise noted.

**Parameter**	**Observations**	**Model results**	**Figure**	**Ref.**
CoB % reduced	0.5%–55%	2%–20%	S1L	(34)
CHO-MFR/MFR	1–10	0.1–2.6	S1A,G	(34)
HS-CoM	4–200 μM	3–200 μM	S1I	(34)
F_{420} % reduced<br>($H_2 \approx 40\ \mu$M)	61%–90%	90%	S1K	(30)
F_{420} % reduced<br>(cell suspension)	17% at $[H_2]$ = 0.7 μM<br>29% at $[H_2]$ = 2 μM<br>50% at $[H_2]$ = 3 μM<br>67% at $[H_2]$ = 7 μM	17% at $[H_2]$ = 7 μM<br>29% at $[H_2]$ = 20 μM<br>50% at $[H_2]$ = 40 μM<br>67% at $[H_2]$ = 80 μM	S1K	(30)

Table S4: The standard redox potentials (E'^0) of reactions that were used to determine $\Delta G_r'^0$ (see table S1).

**Reaction**			E'^0 **(mV)**	**Ref.**
$2H^+ + 2e^-$	$\rightarrow$	$H_{2(aq)}$	−505	(27)
$F_{420} + 2e^-$	$\rightarrow$	$F_{420}H_2$	−360	(32)
CoM-S-S-CoB + $2e^-$	$\rightarrow$	HS-CoB + HS-CoM	−143	(96)
$Fd_{ox} + 2e^-$	$\rightarrow$	Fd_{red}	−400	(27)
CO_2 + MFR + $2e^-$	$\rightarrow$	CHO-MFR + H_2O	−497	(98)

Table S5: Statistics of prior distributions for missing K_M parameters. Each K_M parameter was drawn from a normal distribution with a mean and standard deviation on a logarithmic scale. The prior distributions are based on the observation that K_M values of specific compounds from different enzymes are normally distributed (78). For each compound we compiled the available K_M in the BRENDA database (79), and used their associated distributions. In some cases (K_M for MFR, CHO-H_4MPT, CH_3-H_4MPT, CH_4 and CoM-S-S-CoB) there were insufficient data to generate a prior distribution, and we used instead the distribution of all K_M values (78). The prior distributions were used to estimate the model-observation mismatch, and were weighted by the the inverse of the squared sum of squared errors (1/SSE^2) to generate the posterior distributions (see Methods). The full prior and posterior distributions are in fig. S2.

		**Prior**		**Posterior**
Enzyme	Metabolite(s)	Median (μM)	Standard deviation $\log_{10}(\mu M)$	Median (μM)
Ftr	CHO-H_4MPT, MFR	123[a]	1.24	5
Mch	CHO-H_4MPT	123[a]	1.24	148
Mtd	$F_{420}H_2$	15	0.47	16
Mer	CH_3-H_4MPT	123[a]	1.24	23
Mtr	CH_3-SCoM	692	2.02	560
Mtr	H_4MPT	105	0.60	98
Mcr	CH_4, CoM-SS-CoB	123[a]	1.24	110
Frh	$F_{420}H_2$	15	0.47	11

[a] Distributions that are based on all available K_M measurements (78).

Table S6: Ranges of H_2 concentrations reported in natural environments.

**Environment**	**[H_2] (nM)**	**Ref.**
Freshwater sediments	20	(67, 121)
Marine sediments		
Shallow	0.5–5	(122–124)
Deep	100	(125)
Estuaries	10–100	(126)
Rice paddies	15–60	(127)

Table S7: Estimated environmental csMR based on the relations between measured cell densities and bulk methanogenesis rates in shallow and deep marine sediments.

Source	Max. sediment depth (m)	Temp. (°C)	Cell density (cm^{-3})	bMR (nmol cm^{-3} d^{-1})	csMR (fmol $cell^{-1}$ d^{-1})	Ref.
Hoehler et al., 1994 (W)	0.4	6	4.3×10^{7}	$1.1^{\dagger\,\S}$	2.6×10^{-2}	(128)
Hoehler et al., 1994 (S)	0.4	28	4.3×10^{7}	$32^{\dagger\,\S}$	7.5×10^{-1}	(128)
Claypool et al., 2006	120	10	2.9×10^{5}	7.2×10^{-4}	2.4×10^{-3}	(129)
Sivan et al., 2007	200	10	1.3×10^{5}	2.3×10^{-4}	1.8×10^{-3}	(130)
Parkes et al., 2007	4	16	$5.4\times10^{7\P}$	$4.7\times10^{-1\dagger}$	8.7×10^{-3}	(131)
Beulig et al., 2018	1.4	8	$9.5\times10^{5\P}$	$4.8\times10^{-1\dagger}$	5.0×10^{-1}	(132)
Chuang et al., 2018	40	5	6.8×10^{5}	4.1×10^{-4}	6.0×10^{-4}	(133)
Zhuang et al., 2018‡	6	13	7.9×10^{6}	7.6×10^{-1}	9.5×10^{-2}	(124)
Zhuang et al., 2018$	5	13	7.9×10^{6}	1.0×10^{-3}	1.3×10^{-4}	(124)

Where not measured directly, cell densities were estimated based on their dependence on depth (90, 91) and assuming that of these cells 12% are Archaea in open-ocean sites and 40% in ocean margin sites (92), and that 50% of Archaea are methanogens (91).

† ^{14}C tracer measurements.

§ Tracer measurements were done for both gross methane production and consumption, and the results are shown as a net rate, which is calculated as the difference between the measured forward and backward rates.

¶ Direct cell counts.

‡ Rhone River pro-delta.

$ Gulf of Lion shelf.

Table S8: Physical parameters used in the bio-isotopic model.

**Parameter**	**Value**	**Units**	**Ref.**	**Notes**
Cell volume	2.3	fL/cell	(134)	*M. thermoautotrophicus*
Cell mass	10^{-12}	gdw/cell	(89)	*M. thermoautotrophicus*
Dry weight protein content	41	%	(135)	*M. bryantii*
Membrane thickness	0.5	nm	–	
Diffusivity constant	2.9×10^{-9}	$m^2\ s^{-1}$	(75)	
K_H (H_2)	6.46×10^{-9}	mol L^{-1} Pa^{-1}	(136)	Henry's Law constant at 60°C
K_H (CO_2)	1.42×10^{-7}	mol L^{-1} Pa^{-1}	(136)	Henry's Law constant at 60°C
K_H (CH_4)	7.97×10^{-9}	mol L^{-1} Pa^{-1}	(136)	Henry's Law constant at 60°C
Total ferredoxin (Fd)†	5	mM	–	
Total coenzyme $F_{420}^{\dagger}$	0.5	mM	(35, 135, 137)	
Total coenzyme B (HS-CoB)†	6	mM	(34)	
Total methanofuran (MFR)†	1.8	mM	(34)	

† Total Fd and F_{420} include the oxidized and reduced forms, total coenzyme B includes HS-CoB and CoM-S-S-CoB, and total MFR includes MFR and CHO-MFR. The model is insensitive to initial concentrations of non-coupled metabolites.

REFERENCES AND NOTES

1. M. Saunois, P. Bousquet, B. Poulter, A. Peregon, P. Ciais, J. G. Canadell, E. J. Dlugokencky, G. Etiope, D. Bastviken, S. Houweling, G. Janssens-Maenhout, F. N. Tubiello, S. Castaldi, R. B. Jackson, M. Alexe, V. K. Arora, D. J. Beerling, P. Bergamaschi, D. R. Blake, G. Brailsford, V. Brovkin, L. Bruhwiler, C. Crevoisier, P. Crill, K. Covey, C. Curry, C. Frankenberg, N. Gedney, L. Höglund-Isaksson, M. Ishizawa, A. Ito, F. Joos, H.-S. Kim, T. Kleinen, P. Krummel, J. F. Lamarque, R. Langenfelds, R. Locatelli, T. Machida, S. Maksyutov, K. C. McDonald, J. Marshall, J. R. Melton, I. Morino, V. Naik, S. O'Doherty, F. J. W. Parmentier, P. K. Patra, C. Peng, S. Peng, G. P. Peters, I. Pison, C. Prigent, R. Prinn, M. Ramonet, W. J. Riley, M. Saito, M. Santini, R. Schroeder, I. J. Simpson, R. Spahni, P. Steele, A. Takizawa, B. F. Thornton, H. Tian, Y. Tohjima, N. Viovy, A. Voulgarakis, M. van Weele, G. R. van der Werf, R. Weiss, C. Wiedinmyer, D. J. Wilton, A. Wiltshire, D. Worthy, D. Wunch, X. Xu, Y. Yoshida, B. Zhang, Z. Zhang, Q. Zhu, The global methane budget 2000–2012. *Earth Syst. Sci. Data* **8**, 697–751 (2016).

2. S. Schwietzke, O. A. Sherwood, L. M. P. Bruhwiler, J. B. Miller, G. Etiope, E. J. Dlugokencky, S. E. Michel, V. A. Arling, B. H. Vaughn, J. W. C. White, P. P. Tans, Upward revision of global fossil fuel methane emissions based on isotope database. *Nature* **538**, 88–91 (2016).

3. Z. Lyu, N. Shao, T. Akinyemi, W. B. Whitman, Methanogenesis. *Curr. Biol.* **28**, R727–R732 (2018).

4. M. J. Whiticar, Carbon and hydrogen isotope systematics of bacterial formation and oxidation of methane. *Chem. Geol.* **161**, 291–314 (1999).

5. E. G. Nisbet, E. J. Dlugokencky, M. R. Manning, D. Lowry, R. E. Fisher, J. L. France, S. E. Michel, J. B. Miller, J. W. C. White, B. Vaughn, P. Bousquet, J. A. Pyle, N. J. Warwick, M. Cain, R. Brownlow, G. Zazzeri, M. Lanoisellé, A. C. Manning, E. Gloor, D. E. J. Worthy, E.-G. Brunke, C. Labuschagne, E. W. Wolff, A. L. Ganesan, Rising atmospheric methane: 2007-2014 growth and isotopic shift. *Global Biogeochem. Cycles* **30**, 1356–1370 (2016).

6. K. J. Murata, I. Friedman, M. Cremer, Geochemistry of diagenetic dolomites in Miocene marine formations of California and Oregon (Professional Paper 724-C, United States Geological Survey, 1972).

7. Y. Bottinga, Calculated fractionation factors for carbon and hydrogen isotope exchange in the system calcite-carbon dioxide-graphite-methane-hydrogen-water vapor. *Geochim. Cosmochim. Acta* **33**, 49–64 (1969).

8. L. M. Games, J. M. Hayes, in *Proceedings of the 2nd International Symposium on Environmental Biogeochemistry*, J. O. Nriagu, Ed. (Science Publishers, 1976), vol. 1, pp. 51–73.

9. J. W. Smith, K. W. Gould, D. Rigby, The stable isotope geochemistry of Australian coals. *Org. Geochem.* **3**, 111–131 (1981).

10. L. M. Games, J. Hayes, R. P. Gunsalus, Methane-producing bacteria: Natural fractionations of the stable carbon isotopes. *Geochim. Cosmochim. Acta* **42**, 1295–1297 (1978).

11. G. Fuchs, R. Thauer, H. Ziegler, W. Stichler, Carbon isotope fractionation by *Methanobacterium thermoautotrophicum*. *Arch. Microbiol.* **120**, 135–139 (1979).

12. S. S. Belyaev, R. Wolkin, W. R. Kenealy, M. J. Deniro, S. Epstein, J. G. Zeikus, Methanogenic bacteria from the bondyuzhskoe oil field: General characterization and analysis of stable-carbon isotopic fractionation. *Appl. Environ. Microbiol.* **45**, 691–697 (1983).

13. M. Balabane, E. Galimov, M. Hermann, R. Létolle, Hydrogen and carbon isotope fractionation during experimental production of bacterial methane. *Org. Geochem.* **11**, 115–119 (1987).

14. R. Botz, H. D. Pokojski, M. Schmitt, M. Thomm, Carbon isotope fractionation during bacterial methanogenesis by CO_2 reduction. *Org. Geochem.* **25**, 255–262 (1996).

15. A. M. Zyakun, V. A. Bondar, K. S. Laurinavichus, O. V. Shipin, S. S. Belyaev, M. V. Ivanov, Fractionation of carbon isotopes under the growth of methane-producing bacteria on various substrates. *Mikrobiol. Zhurnal* **50**, 16–22 (1988).

16. D. L. Valentine, A. Chidthaisong, A. Rice, W. S. Reeburgh, S. C. Tyler, Carbon and hydrogen isotope fractionation by moderately thermophilic methanogens. *Geochim. Cosmochim. Acta* **68**, 1571–1590 (2004).

17. H. Penning, C. M. Plugge, P. E. Galand, R. Conrad, Variation of carbon isotope fractionation in hydrogenotrophic methanogenic microbial cultures and environmental samples at different energy status. *Glob. Chang. Biol.* **11**, 2103–2113 (2005).

18. K. Takai, K. Nakamura, T. Toki, U. Tsunogai, M. Miyazaki, J. Miyazaki, H. Hirayama, S. Nakagawa, T. Nunoura, K. Horikoshi, Cell proliferation at 122 degrees C and isotopically heavy CH_4 production by a hyperthermophilic methanogen under high-pressure cultivation. *Proc. Natl. Acad. Sci. U.S.A.* **105**, 10949–10954 (2008).

19. S. Hattori, H. Nashimoto, H. Kimura, K. Koba, K. Yamada, M. Shimizu, H. Watanabe, M. Yoh, N. Yoshida, Hydrogen and carbon isotope fractionation by thermophilic hydrogenotrophic methanogens from a deep aquifer under coculture with fermenters. *Geochem. J.* **46**, 193–200 (2012).

20. T. Okumura, S. Kawagucci, Y. Saito, Y. Matsui, K. Takai, H. Imachi, Hydrogen and carbon isotope systematics in hydrogenotrophic methanogenesis under H_2-limited and H_2-enriched conditions: Implications for the origin of methane and its isotopic diagnosis. *Prog Earth Planet Sci* **3**, 14 (2016).

21. B. D. Topçuoğlu, C. Meydan, T. B. Nguyen, S. Q. Lang, J. F. Holden, Growth kinetics, carbon isotope fractionation, and gene expression in the hyperthermophile

Methanocaldococcus jannaschii during hydrogen-limited growth and interspecies hydrogen transfer. *Appl. Environ. Microbiol.* **85**, 1–14 (2019).

22. D. A. Stolper, A. M. Martini, M. Clog, P. M. Douglas, S. S. Shusta, D. L. Valentine, A. L. Sessions, J. Eiler, Distinguishing and understanding thermogenic and biogenic sources of methane using multiply substituted isotopologues. *Geochim. Cosmochim. Acta* **161**, 219–247 (2015).

23. D. T. Wang, D. S. Gruen, B. S. Lollar, K.-U. Hinrichs, L. C. Stewart, J. F. Holden, A. N. Hristov, J. W. Pohlman, P. L. Morrill, M. Könneke, K. B. Delwiche, E. P. Reeves, C. N. Sutcliffe, D. J. Ritter, J. S. Seewald, J. C. McIntosh, H. F. Hemond, M. D. Kubo, D. Cardace, T. M. Hoehler, S. Ono, Nonequilibrium clumped isotope signals in microbial methane. *Science* **348**, 428–431 (2015).

24. X. Cao, H. Bao, Y. Peng, A kinetic model for isotopologue signatures of methane generated by biotic and abiotic CO_2 methanation. *Geochim. Cosmochim. Acta* **249**, 59–75 (2019).

25. B. A. Wing, I. Halevy, Intracellular metabolite levels shape sulfur isotope fractionation during microbial sulfate respiration. *Proc. Natl. Acad. Sci. U.S.A.* **111**, 18116–18125 (2014).

26. E. Noor, A. Flamholz, W. Liebermeister, A. Bar-Even, R. Milo, A note on the kinetics of enzyme action: A decomposition that highlights thermodynamic effects. *FEBS Lett.* **587**, 2772–2777 (2013).

27. R. A. Alberty, *Thermodynamics of Biochemical Reactions* (John Wiley & Sons Inc., 2003).

28. A.-K. Kaster, J. Moll, K. Parey, R. K. Thauer, Coupling of ferredoxin and heterodisulfide reduction via electron bifurcation in hydrogenotrophic methanogenic archaea. *Proc. Natl. Acad. Sci. U.S.A.* **108**, 2981–2986 (2011).

29. T. Watanabe, O. Pfeil-Gardiner, J. Kahnt, J. Koch, S. Shima, B. J. Murphy, Three-megadalton complex of methanogenic electron-bifurcating and CO_2-fixing enzymes. *Science* **373**, 1151–1156 (2021).

30. L. M. I. de Poorter, W. J. Geerts, J. T. Keltjens, Hydrogen concentrations in methane-forming cells probed by the ratios of reduced and oxidized coenzyme F420. *Microbiology* **151**, 1697–1705 (2005).

31. C. Walsh, Naturally occurring 5-deazaflavin coenzymes: Biological redox roles. *Acc. Chem. Res.* **19**, 216–221 (1986).

32. F. Jacobson, C. Walsh, Properties of 7,8-didemethyl-8-hydroxy-5-deazaflavins relevant to redox coenzyme function in methanogen metabolism. *Biochemistry* **23**, 979–988 (1984).

33. L. D. Eirich, G. D. Vogels, R. S. Wolfe, Distribution of coenzyme F420 and properties of its hydrolytic fragments. *J. Bacteriol.* **140**, 20–27 (1979).

34. L. M. I. de Poorter, W. G. Geerts, A. P. R. Theuvenet, J. T. Keltjens, Bioenergetics of the formyl-methanofuran dehydrogenase and heterodisulfide reductase reactions in *Methanothermobacter thermautotrophicus*. *Eur. J. Biochem.* **270**, 66–75 (2003).

35. P. Vermeij, J. L. Pennings, S. M. Maassen, J. T. Keltjens, G. D. Vogels, Cellular levels of factor 390 and methanogenic enzymes during growth of *Methanobacterium thermoautotrophicum* ΔH. *J. Bacteriol.* **179**, 6640–6648 (1997).

36. J. A. Bassham, G. H. Krause, Free energy changes and metabolic regulation in steady-state photosynthetic carbon reduction. *Biochim. Biophys. Acta BBA - Bioenerg.* **189**, 207–221 (1969).

37. D. A. Beard, H. Qian, Relationship between thermodynamic driving force and one-way fluxes in reversible processes. *PLOS ONE* **2**, e144 (2007).

38. C. Rees, A steady-state model for sulphur isotope fractionation in bacterial reduction processes. *Geochim. Cosmochim. Acta* **37**, 1141–1162 (1973).

39. M. A. Iron, J. Gropp, Cost-effective density functional theory (DFT) calculations of equilibrium isotopic fractionation in large organic molecules. *Phys. Chem. Chem. Phys.* **21**, 17555–17570 (2019).

40. H. Yoshioka, S. Sakata, Y. Kamagata, Hydrogen isotope fractionation by *Methanothermobacter thermoautotrophicus* in coculture and pure culture conditions. *Geochim. Cosmochim. Acta* **72**, 2687–2694 (2008).

41. A. C. Turner, R. Korol, D. L. Eldridge, M. Bill, M. E. Conrad, T. F. Miller, D. A. Stolper, Experimental and theoretical determinations of hydrogen isotopic equilibrium in the system CH_4-H_2-H_2O from 3 to 200°C. *Geochim. Cosmochim. Acta* **314**, 223–269 (2021).

42. S. Scheller, M. Goenrich, R. K. Thauer, B. Jaun, Methyl-coenzyme M reductase from methanogenic archaea: Isotope effects on the formation and anaerobic oxidation of methane. *J. Am. Chem. Soc.* **135**, 14975–14984 (2013).

43. J. Gropp, M. A. Iron, I. Halevy, Theoretical estimates of equilibrium carbon and hydrogen isotope effects in microbial methane production and anaerobic oxidation of methane. *Geochim. Cosmochim. Acta* **295**, 237–264 (2021).

44. H. M. Miller, N. Chaudhry, M. E. Conrad, M. Bill, S. H. Kopf, A. S. Templeton, Large carbon isotope variability during methanogenesis under alkaline conditions. *Geochim. Cosmochim. Acta* **237**, 18–31 (2018).

45. D. B. Nothaft, A. S. Templeton, J. H. Rhim, D. T. Wang, J. Labidi, H. M. Miller, E. S. Boyd, J. M. Matter, S. Ono, E. D. Young, S. H. Kopf, P. B. Kelemen, M. E. Conrad, Geochemical, biological and clumped isotopologue evidence for substantial microbial methane production under carbon limitation in serpentinites of the Samail Ophiolite , Oman. *Eur. J. Vasc. Endovasc. Surg.* **126**, e2020JG006025 (2021).

46. R. K. Thauer, A.-K. Kaster, H. Seedorf, W. Buckel, R. Hedderich, Methanogenic archaea: Ecologically relevant differences in energy conservation. *Nat. Rev. Microbiol.* **6**, 579–591 (2008).

47. S. W. Ragsdale, M. Kumar, Nickel-containing carbon monoxide dehydrogenase/acetyl-CoA synthase. *Chem. Rev.* **96**, 2515–2540 (1996).

48. P. G. Simpson, W. B. Whitman, Anabolic pathways in methanogens, in *Methanogenesis* (Springer, 1993), pp. 445–472.

49. M. J. Alperin, T. M. Hoehler, Anaerobic methane oxidation by archaea/sulfate-reducing bacteria aggregates: 2. Isotopic constraints. *Am. J. Sci.* **309**, 958–984 (2009).

50. K. L. Londry, K. G. Dawson, H. D. Grover, R. E. Summons, A. S. Bradley, Stable carbon isotope fractionation between substrates and products of *Methanosarcina barkeri*. *Org. Geochem.* **39**, 608–621 (2008).

51. T. B. Nguyen, B. D. Topçuoğlu, J. F. Holden, D. E. LaRowe, S. Q. Lang, Lower hydrogen flux leads to larger carbon isotopic fractionation of methane and biomarkers during hydrogenotrophic methanogenesis. *Geochim. Cosmochim. Acta* **271**, 212–226 (2020).

52. S. Kawagucci, M. Kobayashi, S. Hattori, K. Yamada, Y. Ueno, K. Takai, N. Yoshida, Hydrogen isotope systematics among H_2–H_2O–CH_4 during the growth of the hydrogenotrophic methanogen *Methanothermobacter thermautotrophicus* strain ΔH. *Geochim. Cosmochim. Acta* **142**, 601–614 (2014).

53. C. Afting, E. Kremmer, C. Brucker, A. Hochheimer, R. K. Thauer, Regulation of the synthesis of H_2-forming methylenetetrahydromethanopterin dehydrogenase (Hmd) and of HmdII and HmdIII in *Methanothermobacter marburgensis*. *Arch. Microbiol.* **174**, 225–232 (2000).

54. P. M. Vignais, H/D exchange reactions and mechanistic aspects of the hydrogenases. *Coord. Chem. Rev.* **249**, 1677–1690 (2005).

55. S. Walter, S. Laukenmann, A. J. M. Stams, M. K. Vollmer, G. Gleixner, T. Röckmann, The stable isotopic signature of biologically produced molecular hydrogen (H_2). *Biogeosciences* **9**, 4115–4123 (2012).

56. J. H. Rolston, J. Den Hartog, J. P. Butler, The deuterium isotope separation factor between hydrogen and liquid water. *J. Phys. Chem.* **80**, 1064–1067 (1976).

57. D. A. Stolper, M. Lawson, C. L. Davis, A. A. Ferreira, E. V. Santos Neto, G. S. Ellis, M. D. Lewan, A. M. Martini, Y. Tang, M. Schoell, A. L. Sessions, J. M. Eiler, Formation temperatures of thermogenic and biogenic methane. *Science* **344**, 1500–1503 (2014).

58. P. M. J. Douglas, D. A. Stolper, J. M. Eiler, A. L. Sessions, M. Lawson, Y. Shuai, A. Bishop, O. G. Podlaha, A. A. Ferreira, E. V. Santos Neto, M. Niemann, A. S. Steen, L. Huang, L. Chimiak, D. L. Valentine, J. Fiebig, A. J. Luhmann, W. E. Seyfried Jr., G. Etiope, M.

Schoell, W. P. Inskeep, J. J. Moran, N. Kitchen, Methane clumped isotopes: Progress and potential for a new isotopic tracer. *Org. Geochem.* **113**, 262–282 (2017).

59. L. Y. Yeung, Combinatorial effects on clumped isotopes and their significance in biogeochemistry. *Geochim. Cosmochim. Acta* **172**, 22–38 (2016).
60. T. Röckmann, M. E. Popa, M. C. Krol, M. E. G. Hofmann, Statistical clumped isotope signatures. *Sci. Rep.* **6**, 31947 (2016).
61. L. Taenzer, J. Labidi, A. L. Masterson, X. Feng, D. Rumble, E. D. Young, W. D. Leavitt, Low $\Delta^{12}CH_2D_2$ values in microbialgenic methane result from combinatorial isotope effects. *Geochim. Cosmochim. Acta* **285**, 225–236 (2020).
62. J. H. Rhim, "Experimental investigations of isotopologue fractionation during microbial methanogenesis," thesis, Massachusetts Institute of Technology (2020).
63. D. S. Vinson, N. E. Blair, A. M. Martini, S. Larter, W. H. Orem, J. C. McIntosh, Microbial methane from in situ biodegradation of coal and shale: A review and reevaluation of hydrogen and carbon isotope signatures. *Chem. Geol.* **453**, 128–145 (2017).
64. J. J. Jautzy, P. M. J. Douglas, H. Xie, J. M. Eiler, I. D. Clark, CH_4 isotopic ordering records ultra-slow hydrocarbon biodegradation in the deep subsurface. *Earth Planet. Sci. Lett.* **562**, 116841 (2021).
65. N. Zhang, G. T. Snyder, M. Lin, M. Nakagawa, A. Gilbert, N. Yoshida, R. Matsumoto, Y. Sekine, Doubly substituted isotopologues of methane hydrate ($^{13}CH_3D$ and $^{12}CH_2D_2$): Implications for methane clumped isotope effects, source apportionments and global hydrate reservoirs. *Geochim. Cosmochim. Acta* **315**, 127–151 (2021).
66. J. L. Pennings, P. Vermeij, L. M. de Poorter, J. T. Keltjens, G. D. Vogels, Adaptation of methane formation and enzyme contents during growth of *Methanobacterium thermoautotrophicum* (strain ΔH) in a fed-batch fermentor. *Antonie Van Leeuwenhoek* **77**, 281–291 (2000).
67. R. Conrad, T. J. Phelps, J. G. Zeikus, Gas metabolism evidence in support of the juxtaposition of hydrogen-producing and methanogenic bacteria in sewage sludge and lake sediments. *Appl. Environ. Microbiol.* **50**, 595–601 (1985).
68. E. Giraldo-Gomez, S. Goodwin, M. S. Switzenbaum, Influence of mass transfer limitations on determination of the half saturation constant for hydrogen uptake in a mixed-culture CH_4-producing enrichment. *Biotechnol. Bioeng.* **40**, 768–776 (1992).
69. Y.-S. Lin, V. B. Heuer, T. Goldhammer, M. Y. Kellermann, M. Zabel, K.-U. Hinrichs, Towards constraining H_2 concentration in subseafloor sediment: A proposal for combined analysis by two distinct approaches. *Geochim. Cosmochim. Acta* **77**, 186–201 (2012).
70. J. A. Bradley, S. Arndt, J. P. Amend, E. Burwicz, A. W. Dale, M. Egger, D. E. LaRowe, Widespread energy limitation to life in global subseafloor sediments. *Sci. Adv.* **6**, eaba0697 (2020).

71. C. K. Paull, T. D. Lorenson, W. S. Borowski, W. Ussler III, K. Olsen, N. M. Rodriguez, Isotopic composition of CH_4, CO_2 species, and sedimentary organic matter within samples from the Blake Ridge: Gas source implications. *Proc. Ocean Drill. Program Sci. Results* **164**, 67–78 (2000).

72. J. W. Pohlman, M. Kaneko, V. B. Heuer, R. B. Coffin, M. Whiticar, Methane sources and production in the northern Cascadia margin gas hydrate system. *Earth Planet. Sci. Lett.* **287**, 504–512 (2009).

73. G. Wegener, J. Gropp, H. Taubner, I. Halevy, M. Elvert, Sulfate-dependent reversibility of intracellular reactions explains the opposing isotope effects in the anaerobic oxidation of methane. *Sci. Adv.* **7**, eabe4939 (2021).

74. M. K. Lloyd, E. Trembath-Reichert, K. S. Dawson, S. J. Feakins, M. Mastalerz, V. J. Orphan, A. L. Sessions, J. M. Eiler, Methoxyl stable isotopic constraints on the origins and limits of coal-bed methane. *Science* **374**, 894–897 (2021).

75. A. Missner, P. Kügler, S. M. Saparov, K. Sommer, J. C. Mathai, M. L. Zeidel, P. Pohl, Carbon dioxide transport through membranes. *J. Biol. Chem.* **283**, 25340–25347 (2008).

76. U. Deppenmeier, V. Müller, Life close to the thermodynamic limit: How methanogenic archaea conserve energy. *Results Probl. Cell Differ.* **45**, 123–152 (2008).

77. A. Flamholz, E. Noor, A. Bar-Even, R. Milo, eQuilibrator—The biochemical thermodynamics calculator. *Nucleic Acids Res.* **40**, D770–D775 (2012).

78. A. Bar-Even, E. Noor, Y. Savir, W. Liebermeister, D. Davidi, D. S. Tawfik, R. Milo, The moderately efficient enzyme: Evolutionary and physicochemical trends shaping enzyme parameters. *Biochemistry* **50**, 4402–4410 (2011).

79. A. Chang, L. Jeske, S. Ulbrich, J. Hofmann, J. Koblitz, I. Schomburg, M. Neumann-Schaal, D. Jahn, D. Schomburg, BRENDA, the ELIXIR core data resource in 2021: New developments and updates. *Nucleic Acids Res.* **49**, D498–D508 (2021).

80. J. K. Hobbs, W. Jiao, A. D. Easter, E. J. Parker, L. A. Schipper, V. L. Arcus, Change in heat capacity for enzyme catalysis determines temperature dependence of enzyme catalyzed rates. *ACS Chem. Biol.* **8**, 2388–2393 (2013).

81. L. A. Schipper, J. K. Hobbs, S. Rutledge, V. L. Arcus, Thermodynamic theory explains the temperature optima of soil microbial processes and high Q10 values at low temperatures. *Glob. Chang. Biol.* **20**, 3578–3586 (2014).

82. J. Gropp, M. A. Iron, I. Halevy, Corrigendum to “Theoretical estimates of equilibrium carbon and hydrogen isotope effects in microbial methane production and anaerobic oxidation of methane” [Geochim. Cosmochim. Acta 295 (2021) 237–264]. *Geochim. Cosmochim. Acta* **306**, 386–389 (2021).

83. A. Sattler, Hydrogen/deuterium (H/D) exchange catalysis in alkanes. *ACS Catal.* **8**, 2296–2312 (2018).

84. M. Gómez-Gallego, M. A. Sierra, Kinetic isotope effects in the study of organometallic reaction mechanisms. *Chem. Rev.* **111**, 4857–963 (2011).

85. E. L. Hendrickson, A. K. Haydock, B. C. Moore, W. B. Whitman, J. A. Leigh, Functionally distinct genes regulated by hydrogen limitation and growth rate in methanogenic Archaea. *Proc. Natl. Acad. Sci. U.S.A.* **104**, 8930–8934 (2007).

86. S. Kato, T. Kosaka, K. Watanabe, Comparative transcriptome analysis of responses of *Methanothermobacter thermautotrophicus* to different environmental stimuli. *Environ. Microbiol.* **10**, 893–905 (2008).

87. L. G. Bonacker, S. Baudner, R. K. Thauer, Differential expression of the two methyl-coenzyme M reductases in *Methanobacterium thermoautotrophicum* as determined immunochemically via isoenzyme-specific antisera. *Eur. J. Biochem.* **206**, 87–92 (1992).

88. L. G. Bonacker, S. Baudner, E. Mörschel, R. Böcher, R. K. Thauer, Properties of the two isoenzymes of methyl-coenzyme M reductase in *Methanobacterium thermoautotrophicum*. *Eur. J. Biochem.* **217**, 587–95 (1993).

89. M. A. Lever, K. L. Rogers, K. G. Lloyd, J. Overmann, B. Schink, R. K. Thauer, T. M. Hoehler, B. B. Jørgensen, Life under extreme energy limitation: A synthesis of laboratory- and field-based investigations. *FEMS Microbiol. Rev.* **39**, 688–728 (2015).

90. J. Kallmeyer, R. Pockalny, R. R. Adhikari, D. C. Smith, S. D'Hondt, Global distribution of microbial abundance and biomass in subseafloor sediment. *Proc. Natl. Acad. Sci. U.S.A.* **109**, 16213–16216 (2012).

91. C. Petro, P. Starnawski, A. Schramm, K. U. Kjeldsen, Microbial community assembly in marine sediments. *Aquat. Microb. Ecol.* **79**, 177–195 (2017).

92. T. Hoshino, F. Inagaki, Abundance and distribution of Archaea in the subseafloor sedimentary biosphere. *ISME J.* **13**, 227–231 (2019).

93. P. A. Bertram, R. K. Thauer, Thermodynamics of the formylmethanofuran dehydrogenase reaction in *Methanobacterium Thermoautotrophicum*. *Eur. J. Biochem.* **226**, 811–818 (1994).

94. W. Buckel, R. K. Thauer, Energy conservation via electron bifurcating ferredoxin reduction and proton/Na^+ translocating ferredoxin oxidation. *Biochim. Biophys. Acta* **1827**, 94–113 (2013).

95. B. E. Maden, Tetrahydrofolate and tetrahydromethanopterin compared: Functionally distinct carriers in C1 metabolism. *Biochem. J.* **350** Pt 3, 609–629 (2000).

96. M. Tietze, A. Beuchle, I. Lamla, N. Orth, M. Dehler, G. Greiner, U. Beifuss, Redox potentials of Methanophenazine and CoB-S-S-CoM, factors involved in electron transport in methanogenic archaea. *Chembiochem* **4**, 333–335 (2003).

97. G. Börner, M. Karrasch, R. K. Thauer, Molybdopterin adenine dinucleotide and molybdopterin hypoxanthine dinucleotide in formylmethanofuran dehydrogenase from *Methanobacterium thermoautotrophicum* (Marburg). *FEBS Lett.* **290**, 31–34 (1991).

98. P. A. Bertram, M. Karrasch, R. A. Schmitz, R. Bocher, S. P. J. Albracht, R. K. Thauer, Formylmethanofuran dehydrogenases from methanogenic Archaea. Substrate specificity, EPR properties and reversible inactivation by cyanide of the molybdenum or tungsten iron-sulfur proteins. *Eur. J. Biochem.* **220**, 477–484 (1994).

99. M. I. Donnelly, R. S. Wolfe, The role of formylmethanofuran: Tetrahydromethanopterin formyltransferase in methanogenesis from carbon dioxide. *J. Biol. Chem.* **261**, 16653–16659 (1986).

100. J. Breitung, R. K. Thauer, Formylmethanofuran: Tetrahydromethanopterin formyltransferase from *Methanosarcina barkeri*. Identification of N5-formyltetrahydromethanopterin as the product. *FEBS Lett.* **275**, 226–230 (1990).

101. A. A. DiMarco, M. I. Donnelly, R. S. Wolfe, Purification and properties of the 5,10-methenyltetrahydromethanopterin cyclohydrolase from *Methanobacterium thermoautotrophicum*. *J. Bacteriol.* **168**, 1372–1377 (1986).

102. J. Breitung, R. A. Schmitz, K. O. Stetter, R. K. Thauer, N^5,N^{10}-Methenyltetrahydromethanopterin cyclohydrolase from the extreme thermophile *Methanopyrus kandleri*: Increase of catalytic efficiency (k_{cat}/K_M) and thermostability in the presence of salts. *Arch. Microbiol.* **156**, 517–524 (1991).

103. B. W. te Brömmelstroet, C. M. Hensgens, W. J. Geerts, J. T. Keltjens, C. van der Drift, G. D. Vogels, Purification and properties of ^{5,10}-methenyltetrahydromethanopterin cyclohydrolase from *Methanosarcina barkeri*. *J. Bacteriol.* **172**, 564–571 (1990).

104. B. Mukhopadhyay, L. Daniels, Aerobic purification of N5,N10-methylenetetrahydromethanopterin dehydrogenase, separated from N^5,N^{10}-methylenetetrahydromethanopterin cyclohydrolase, from *Methanobacterium thermoautotrophicum* strain Marburg. *Can. J. Microbiol.* **35**, 499–507 (1989).

105. B. te Brömmelstroet, C. M. Hensgens, J. T. Keltjens, C. van der Drift, G. D. Vogels, Purification and characterization of coenzyme F_{420}-dependent ^{5,10}-methylenetetrahydromethanopterin dehydrogenase from *Methanobacterium thermoautotrophicum* strain ΔH. *Biochim. Biophys. Acta Gen. Subj.* **1073**, 77–84 (1991).

106. C. Zirngibl, W. Dongen, B. Schworer, R. Bunau, M. Richter, A. Klein, R. K. Thauer, H_2-forming methylenetetrahydromethanopterin dehydrogenase, a novel type of hydrogenase without iron-sulfur clusters in methanogenic archaea. *Eur. J. Biochem.* **208**, 511–520 (1992).

107. E. J. Lyon, S. Shima, G. Buurman, S. Chowdhuri, A. Batschauer, K. Steinbach, R. K. Thauer, UV-A/blue-light inactivation of the ‘metal-free’ hydrogenase (Hmd) from methanogenic archaea. *Eur. J. Biochem.* **271**, 195–204 (2004).

108. G. C. Hartmann, A. R. Klein, M. Linder, R. K. Thauer, Purification, properties and primary structure of H2-forming N^5,N^{10}-methylenetetrahydromethanopterin dehydrogenase from *Methanococcus thermolithotrophicus*. *Arch. Microbiol.* **165**, 187–193 (1996).

109. K. Ma, R. K. Thauer, Purification and properties of N5, N10-methylenetetrahydromethanopterin reductase from *Methanobacterium thermoautotrophicum* (strain Marburg). *Eur. J. Biochem.* **191**, 187–193 (1990).

110. B. W. te Brömmelstroet, W. J. Geerts, J. T. Keltjens, C. van der Drift, G. D. Vogels, C. van der Drift, G. D. Vogels, Purification and properties of 5,10-methylenetetrahydromethanopterin dehydrogenase and 5,10-methylenetetrahydromethanopterin reductase, two coenzyme F_{420}-dependent enzymes, from *Methanosarcina barkeri*. *Biochim. Biophys. Acta Protein Struct. Mol. Enzymol.* **1079**, 293–302 (1991).

111. T. Lienard, B. Becher, M. Marschall, S. Bowien, G. Gottschalk, Sodium ion translocation by N^5-methyltetrahydromethanopterin: Coenzyme M methyltransferase from *Methanosarcina mazei* Gö1 reconstituted in ether lipid liposomes. *Eur. J. Biochem.* **239**, 857–864 (1996).

112. V. R. Vepachedu, J. G. Ferry, Role of the fused corrinoid/methyl transfer protein CmtA during CO-dependent growth of *Methanosarcina acetivorans*. *J. Bacteriol.* **194**, 4161–4168 (2012).

113. S. Rospert, R. Böcher, S. P. Albracht, R. K. Thauer, Methyl-coenzyme M reductase preparations with high specific activity from H_2-preincubated cells of *Methanobacterium thermoautotrophicum*. *FEBS Lett.* **291**, 371–375 (1991).

114. M. Dey, X. Li, R. C. Kunz, S. W. Ragsdale, Detection of organometallic and radical intermediates in the catalytic mechanism of methyl-coenzyme M reductase using the natural substrate methyl-coenzyme M and a coenzyme B substrate analogue. *Biochemistry* **49**, 10902–10911 (2010).

115. P. E. Rouvière, T. A. Bobik, R. S. Wolfe, Reductive activation of the methyl coenzyme M methylreductase system of *Methanobacterium thermoautotrophicum* delta H. *J. Bacteriol.* **170**, 3946–3952 (1988).

116. J. Ellermann, S. Rospert, R. K. Thauer, M. Bokranz, A. Klein, M. Voges, A. Berkessel, Methyl-coenzyme-M reductase from *Methanobacterium thermoautotrophicum* (strain Marburg). Purity, activity and novel inhibitors. *Eur. J. Biochem.* **184**, 63–68 (1989).

117. J. A. Fox, D. J. Livingston, W. H. Orme-Johnson, C. T. Walsh, 8-Hydroxy-5-deazaflavin-reducing hydrogenase from *Methanobacterium thermoautotrophicum*: 1. Purification and characterization. *Biochemistry* **26**, 4219–4227 (1987).

118. E. Setzke, R. Hedderich, S. Heiden, R. K. Thauer, H_2: Heterodisulfide oxidoreductase complex from *Methanobacterium thermoautotrophicum*. Composition and properties. *Eur. J. Biochem. FEBS* **220**, 139–148 (1994).

119. J. Meuer, S. Bartoschek, J. Koch, A. Kunkel, R. Hedderich, Purification and catalytic properties of Ech hydrogenase from *Methanosarcina barkeri*. *Eur. J. Biochem.* **265**, 325–335 (1999).

120. R. Hedderich, A. Berkessel, R. K. Thauer, Purification and properties of heterodisulfide reductase from *Methanobacterium thermoautotrophicum* (strain Marburg). *Eur. J. Biochem.* **193**, 255–261 (1990).

121. D. R. Lovley, S. Goodwin, Hydrogen concentrations as an indicator of the predominant terminal electron-accepting reactions in aquatic sediments. *Geochim. Cosmochim. Acta* **52**, 2993–3003 (1988).

122. P. C. Novelli, A. R. Michelson, M. I. Scranton, G. T. Banta, J. E. Hobbie, R. W. Howarth, Hydrogen and acetate cycling in two sulfate-reducing sediments: Buzzards Bay and Town Cove, Mass. *Geochim. Cosmochim. Acta* **52**, 2477–2486 (1988).

123. T. M. Hoehler, M. J. Alperin, D. B. Albert, C. S. Martens, Thermodynamic control on hydrogen concentrations in anoxic sediments. *Geochim. Cosmochim. Acta* **62**, 1745–1756 (1998).

124. G.-C. Zhuang, V. B. Heuer, C. S. Lazar, T. Goldhammer, J. Wendt, V. A. Samarkin, M. Elvert, A. P. Teske, S. B. Joye, K.-U. Hinrichs, Relative importance of methylotrophic methanogenesis in sediments of the Western Mediterranean Sea. *Geochim. Cosmochim. Acta* **224**, 171–186 (2018).

125. A. Ijiri, F. Inagaki, Y. Kubo, R. R. Adhikari, S. Hattori, T. Hoshino, H. Imachi, S. Kawagucci, Y. Morono, Y. Ohtomo, S. Ono, S. Sakai, K. Takai, T. Toki, D. T. Wang, M. Y. Yoshinaga, G. L. Arnold, J. Ashi, D. H. Case, T. Feseker, K.-U. Hinrichs, Y. Ikegawa, M. Ikehara, J. Kallmeyer, H. Kumagai, M. A. Lever, S. Morita, K.-I. Nakamura, Y. Nakamura, M. Nishizawa, V. J. Orphan, H. Røy, F. Schmidt, A. Tani, W. Tanikawa, T. Terada, H. Tomaru, T. Tsuji, U. Tsunogai, Y. T. Yamaguchi, N. Yoshida, Deep-biosphere methane production stimulated by geofluids in the Nankai accretionary complex. *Sci. Adv.* **4**, eaao4631 (2018).

126. R. H. Michener, M. I. Scranton, P. Novelli, Hydrogen (H_2) distributions in the carmans river estuary. *Estuar. Coast. Shelf Sci.* **27**, 223–235 (1988).

127. H. Yao, R. Conrad, Thermodynamics of methane production in different rice paddy soils from China, the Philippines and Italy. *Soil Biol. Biochem.* **31**, 463–473 (1999).

128. T. M. Hoehler, M. J. Alperin, D. B. Albert, C. S. Martens, Field and laboratory studies of methane oxidation in an anoxic marine sediment: Evidence for a methanogen-sulfate reducer consortium. *Global Biogeochem. Cycles* **8**, 451–463 (1994).

129. G. E. Claypool, A. V. Milkov, Y. J. Lee, M. E. Torres, W. S. Borowski, H. Tomaru, Microbial methane generation and gas transport in shallow sediments of an accretionary complex, southern Hydrate Ridge (ODP Leg 204), offshore Oregon, USA. *Proc. Ocean Drill. Program Sci. Results* **204**, 1–52 (2006).

130. O. Sivan, D. P. Schrag, R. W. Murray, Rates of methanogenesis and methanotrophy in deep-sea sediments. *Geobiology* **5**, 141–151 (2007).

131. R. J. Parkes, B. A. Cragg, N. Banning, F. Brock, G. Webster, J. C. Fry, E. Hornibrook, R. D. Pancost, S. Kelly, N. Knab, B. B. Jørgensen, J. Rinna, A. J. Weightman, Biogeochemistry and biodiversity of methane cycling in subsurface marine sediments (Skagerrak, Denmark). *Environ. Microbiol.* **9**, 1146–1161 (2007).

132. F. Beulig, H. Røy, S. E. McGlynn, B. B. Jørgensen, Cryptic CH_4 cycling in the sulfate–methane transition of marine sediments apparently mediated by ANME-1 archaea. *ISME J.* **13**, 250–262 (2018).

133. P.-C. Chuang, T. Frank Yang, K. Wallmann, R. Matsumoto, C.-Y. Hu, H.-W. Chen, S. Lin, C.-H. Sun, H.-C. Li, Y. Wang, A. W. Dale, Carbon isotope exchange during anaerobic oxidation of methane (AOM) in sediments of the northeastern South China Sea. *Geochim. Cosmochim. Acta* **246**, 138–155 (2018).

134. P. Schönheit, H. J. Perski, ATP synthesis driven by a potassium diffusion potential in *Methanobacterium thermoautotrophicum* is stimulated by sodium. *FEMS Microbiol. Lett.* **20**, 263–267 (1983).

135. E. Heine-Dobbernack, S. M. Schoberth, H. Sahm, Relationship of intracellular coenzyme F_{420} content to growth and metabolic activity of *Methanobacterium bryantii* and *Methanosarcina barkeri*. *Appl. Environ. Microbiol.* **54**, 454–459 (1988).

136. R. Sander, Compilation of Henry's law constants (version 4.0) for water as solvent *Atmos. Chem. Phys.* **15**, 4399–4981 (2015).

137. A. M. Feist, J. C. M. Scholten, B. Ø. Palsson, F. J. Brockman, T. Ideker, Modeling methanogenesis with a genome-scale metabolic reconstruction of *Methanosarcina barkeri*. *Mol. Syst. Biol.* **2**, 2006.0004 (2006).

138. M. Whiticar, E. Faber, M. Schoell, Biogenic methane formation in marine and freshwater environments: CO_2 reduction *vs.* acetate fermentation—Isotope evidence. *Geochim. Cosmochim. Acta* **50**, 693–709 (1986).

139. J. McIntosh, A. Martini, S. Petsch, R. Huang, K. Nüsslein, Biogeochemistry of the Forest City Basin coalbed methane play. *Int. J. Coal Geol.* **76**, 111–118 (2008).

140. M. F. Kirk, B. H. Wilson, K. A. Marquart, L. H. Zeglin, D. S. Vinson, T. M. Flynn, Solute concentrations influence microbial methanogenesis in coal-bearing strata of the Cherokee basin, USA. *Front. Microbiol.* **6**, 1287 (2015).

141. J. C. McIntosh, L. M. Walter, A. M. Martini, Pleistocene recharge to midcontinent basins: Effects on salinity structure and microbial gas generation. *Geochim. Cosmochim. Acta* **66**, 1681–1700 (2002).

142. D. Strapoć, M. Mastalerz, A. Schimmelmann, A. Drobniak, S. Hedges, Variability of geochemical properties in a microbially dominated coalbed gas system from the eastern margin of the Illinois Basin, USA. *Int. J. Coal Geol.* **76**, 98–110 (2008).

143. M. E. Schlegel, J. C. McIntosh, B. L. Bates, M. F. Kirk, A. M. Martini, Comparison of fluid geochemistry and microbiology of multiple organic-rich reservoirs in the Illinois Basin, USA: Evidence for controls on methanogenesis and microbial transport. *Geochim. Cosmochim. Acta* **75**, 1903–1919 (2011).

144. R. M. Flores, C. A. Rice, G. D. Stricker, A. Warden, M. S. Ellis, Methanogenic pathways of coal-bed gas in the Powder River Basin, United States: The geologic factor. *Int. J. Coal Geol.* **76**, 52–75 (2008).

145. B. L. Bates, J. C. McIntosh, K. A. Lohse, P. D. Brooks, Influence of groundwater flowpaths, residence times and nutrients on the extent of microbial methanogenesis in coal beds: Powder River Basin, USA. *Chem. Geol.* **284**, 45–61 (2011).

146. K. A. Baublys, S. K. Hamilton, S. D. Golding, S. Vink, J. Esterle, Microbial controls on the origin and evolution of coal seam gases and production waters of the Walloon Subgroup; Surat Basin, Australia. *Int. J. Coal Geol.* **147-148**, 85–104 (2015).

147. R. Aravena, S. Harrison, J. Barker, H. Abercrombie, D. Rudolph, Origin of methane in the Elk Valley coalfield, southeastern British Columbia, Canada. *Chem. Geol.* **195**, 219–227 (2003).

148. N. Riedinger, M. J. Formolo, T. W. Lyons, S. Henkel, A. Beck, S. Kasten, An inorganic geochemical argument for coupled anaerobic oxidation of methane and iron reduction in marine sediments. *Geobiology* **12**, 172–181 (2014).

149. T. Giunta, E. D. Young, O. Warr, I. Kohl, J. L. Ash, A. Martini, S. O. Mundle, D. Rumble, I. Pérez-Rodríguez, M. Wasley, D. E. LaRowe, A. Gilbert, B. Sherwood Lollar, Methane sources and sinks in continental sedimentary systems: New insights from paired clumped isotopologues $^{13}CH_3D$ and $^{12}CH_2D_2$. *Geochim. Cosmochim. Acta* **245**, 327–351 (2019).

150. T. Giunta, J. Labidi, I. E. Kohl, L. Ruffine, J. P. Donval, L. Géli, M. N. Çağatay, H. Lu, E. D. Young, Evidence for methane isotopic bond re-ordering in gas reservoirs sourcing cold seeps from the Sea of Marmara. *Earth Planet. Sci. Lett.* **553**, 116619 (2021).

151. D. S. Vinson, N. E. Blair, D. J. Ritter, A. M. Martini, J. C. McIntosh, Carbon mass balance, isotopic tracers of biogenic methane, and the role of acetate in coal beds: Powder River Basin (USA). *Chem. Geol.* **530**, 119329 (2019).

152. H. Schweitzer, D. Ritter, J. McIntosh, E. Barnhart, A. B. Cunningham, D. Vinson, W. Orem, M. W. Fields, Changes in microbial communities and associated water and gas geochemistry across a sulfate gradient in coal beds: Powder River Basin, USA. *Geochim. Cosmochim. Acta* **245**, 495–513 (2019).

153. A. M. Martini, L. M. Walter, J. M. Budai, T. C. W. Ku, C. J. Kaiser, M. Schoell, Genetic and temporal relations between formation waters and biogenic methane: Upper Devonian Antrim Shale, Michigan Basin, USA. *Geochim. Cosmochim. Acta* **62**, 1699–1720 (1998).

154. A. M. Martini, L. M. Walter, T. C. W. Ku, J. M. Budai, J. C. McIntosh, M. Schoell, Microbial production and modification of gases in sedimentary basins: A geochemical case study from a Devonian shale gas play, Michigan basin. *AAPG Bull.* **87**, 1355–1375 (2003).

155. P. D. Jenden, I. R. Kaplan, Comparison of microbial* gases from the Middle America Trench and Scripps Submarine Canyon: Implications for the origin of natural gas. *Appl. Geochem.* **1**, 631–646 (1986).

156. U. Berner, M. Von Breymann, E. Faber, P. Bertrand, in *Bacterial Gas*, R. Vially, Ed. (Editions Technip, Paris, 1992), pp. 147–156.
