# Supplementary material for: Controls on the isotopic composition of microbial methane
Source: Sci Adv. 2022 Apr 6;8(14):eabm5713. doi: 10.1126/sciadv.abm5713 (PMC8985922; doi:10.1126/sciadv.abm5713)
Supplement: Supplementary file 3 — Data S1 [file sciadv.abm5713_data_file_s1.zip › readme.rtf]

@authors: Jonathan Gropp, Qusheng Jin, Itay HalevyThis folder contains Matlab scripts and functions that are required to run the metabolic-isotopic model of hydrogenotrophic methanogenesis that is described in the manuscript “Controls on the isotopic composition of microbial methane”, DOI: 10.1101/2021.09.14.460204.All these files are required to run the model, and should be in the same folder.Files included in this folder:Scripts*******run_bioiso_model.m    - Main file to run the code.metModel_param.m      - Thermodynamic and kinetic parameters of the metabolic model.metModel_ratelaw.m    - Calculates the gross forward and reverse rates of reactions.Functions*********metModel_main.m       - Main function of the metabolic model.metModel_ODEsol.m     - Ordinary Differential Equations (ODEs) solver for the metabolic model.isoModelCarb.m        - Bulk carbon isotopic model.isoModelHydr.m        - Bulk hydrogen isotopic model.isoModelClumped_13D.m - Clumped model for 13C—D substituted isotopologues.isoModelClumped_DD.m  - Clumped model for doubly 12C—D substituted isotopologues.calculate_dGr.m       - Calculate the actual transformed Gibbs free energy a the reaction.calc_EFFs.m           - Calculate the temperature dependent equilibrium fractionation factors (EFFs)..mat file*********KFF_distributions.mat - Matrices for posterior kinetic fractionations factors (KFFs).
